# Quantitative bone marrow lesion size in osteoarthritic knees correlates with cartilage damage and predicts longitudinal cartilage loss

**DOI:** 10.1186/1471-2474-12-217

**Published:** 2011-09-30

**Authors:** Jeffrey B Driban, Grace H Lo, Ji Yeon Lee, Robert J Ward, Eric Miller, Jincheng Pang, Lori Lyn Price, Timothy E McAlindon

**Affiliations:** 1 Division of Rheumatology, Tufts Medical Center, 800 Washington Street, Box #406, Boston, MA 02111, USA; 2 Section of Immunology, Allergy, and Rheumatology, Michael E. DeBakey VAMC, and Baylor College of Medicine, 1 Baylor Plaza, BCM-285, Houston, TX 77030, USA; 3 Department of Radiology, Tufts Medical Center, 800 Washington Street, Box #299, Boston, MA 02111, USA; 4 Department of Electrical and Computer Engineering, Tufts University, 216 Halligan Hall, Medford, MA 02155, USA; 5 Biostatistics Research Center, Institute for Clinical Research and Health Policy Studies, Tufts Medical Center, 800 Washington Street, Box #63, Boston, MA 02111, USA

## Abstract

**Background:**

Bone marrow lesions (BMLs), common osteoarthritis-related magnetic resonance imaging findings, are associated with osteoarthritis progression and pain. However, there are no articles describing the use of 3-dimensional quantitative assessments to explore the longitudinal relationship between BMLs and hyaline cartilage loss. The purpose of this study was to assess the cross-sectional and longitudinal descriptive characteristics of BMLs with a simple measurement of approximate BML volume, and describe the cross-sectional and longitudinal relationships between BML size and the extent of hyaline cartilage damage.

**Methods:**

107 participants with baseline and 24-month follow-up magnetic resonance images from a clinical trial were included with symptomatic knee osteoarthritis. An 'index' compartment was identified for each knee defined as the tibiofemoral compartment with greater disease severity. Subsequently, each knee was evaluated in four regions: index femur, index tibia, non-index femur, and non-index tibia. Approximate BML volume, the product of three linear measurements, was calculated for each BML within a region. Cartilage parameters in the index tibia and femur were measured based on manual segmentation.

**Results:**

BML volume changes by region were: index femur (median [95% confidence interval of the median]) 0.1 cm^3 ^(-0.5 to 0.9 cm^3^), index tibia 0.5 cm^3 ^(-0.3 to 1.7 cm^3^), non-index femur 0.4 cm^3 ^(-0.2 to 1.6 cm^3^), and non-index tibia 0.2 cm^3 ^(-0.1 to 1.2 cm^3^). Among 44 knees with full thickness cartilage loss, baseline tibia BML volume correlated with baseline tibia full thickness cartilage lesion area (*r *= 0.63, *p*< 0.002) and baseline femur BML volume with longitudinal change in femoral full thickness cartilage lesion area (*r *= 0.48 *p*< 0.002).

**Conclusions:**

Many regions had no or small longitudinal changes in approximate BML volume but some knees experienced large changes. Baseline BML size was associated to longitudinal changes in area of full thickness cartilage loss.

## Background

Knee osteoarthritis (OA) is the most common form of arthritis, accounting for substantial disability in the general population [1]. However, there are many gaps in our knowledge regarding the pathophysiology of this disease. Recent evidence suggests that peri-articular bone changes are integral to knee OA pathophysiology and may be important for identifying new OA treatments [2-13].

**Figure 1:**
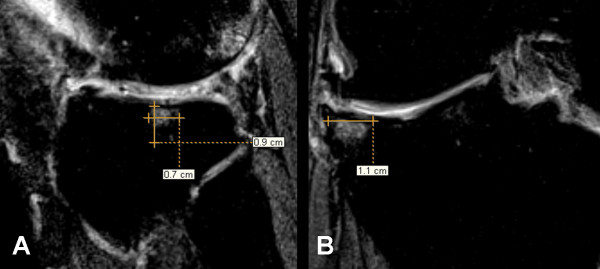
**Bone marrow lesion measurements within the lateral tibia**. a. In the sagittal image the superior-inferior dimension (0.9 mm) and anterior-posterior dimension (0.7 mm) are measured. b. In the coronal image the medial-lateral dimension is measured (1.1 mm). The image contrast is optimized for measuring bone marrow lesions based on the measurement protocol (not for viewing other anatomical structures).

Bone marrow lesions (BMLs), common OA-related magnetic resonance (MR) imaging findings (Figure 1), are associated with OA progression and pain [2-13]. BMLs have been implicated with increased odds of hyaline cartilage damage and subchondral bone attrition [2-5,7-9,12]. To date, no one has published articles describing the use of 3-dimensional quantitative assessments to explore the longitudinal relationship between BMLs and hyaline cartilage.

The purpose of this study was to use a simple method of measuring approximate BML volume that uses the product of three linear measurements across images. This method was selected because it does not require proprietary software, requires less time to measure, and provides a continuous measure of approximate BML size. In this manuscript we 1) report cross-sectional and longitudinal descriptive BML characteristics with this new method, and 2) describe the cross-sectional and longitudinal relationships between approximate BML volume and the extent of hyaline cartilage damage using quantitative 3-dimensional measurements.

## Methods

### Study sample

This study was a secondary analysis of a recently completed randomized, placebo-controlled, clinical trial of vitamin D among participants with knee OA (*n *= 146). All participants with both baseline and 24-month follow-up images were included in this study (*n *= 107 participants). All participants were required to have knee OA as defined by the American College of Rheumatology [14] and be over 45 years of age. Additional inclusion criteria were that participants had to have chronic knee discomfort (knee discomfort on most days for at least one month in past 12 months), Western Ontario and McMaster Universities Osteoarthritis Index (WOMAC) pain subscale ≥ 1, tibiofemoral OA on posterior-anterior weight-bearing semi-flexed knee radiographs (equivalent to Kellgren and Lawrence [KL] grade ≥ 2), and clinical examination confirming knee pain or discomfort referable to the knee joint. To compare the cross-sectional and longitudinal relationships between regional approximate BML volume and the extent of hyaline cartilage damage a sample consisting of 44 knees was selected that only included knees with full thickness cartilage lesions at baseline. This sample is smaller than the full sample because these analyses were restricted to knees with full thickness cartilage lesions to avoid statistical issues associated with more than half of the knees having no areas of full thickness cartilage loss. The Institutional Review Board of Tufts Medical Center approved the study and informed consent was obtained from all participants prior to inclusion.

### Knee selection

One knee was identified as the study knee for each participant. If both knees were eligible, then the knee with a greater WOMAC pain subscale score was selected. If both knees had equivalent WOMAC pain subscale scores, the knee with a greater KL grade was chosen. Finally, if WOMAC pain subscale scores and KL grades were equivalent for both knees, the study knee was randomly selected.

### Index compartment selection

Within each knee, a rheumatologist (TEM) defined an index compartment (medial or lateral tibiofemoral) as the compartment with greater pathology based on radiographs. If the medial and lateral tibiofemoral compartments were comparable then MR images were assessed for cartilage damage, bone marrow lesions, and meniscal damage. A second rheumatologist (JYL) independently verified the index compartment by evaluating MR images. If disagreement arose a consensus decision was achieved.

### MR image acquisition

MR images of the study knees were obtained at baseline and at 2 years follow-up on a Siemens Magnetom Avanto 1.5T (Malvern, PA). The sequences of relevance for BML assessment were sagittal and coronal intermediate-weighted (IW) fat-suppressed (FS) images with time to recovery (TR) of 2950 ms, time to echo (TE) of 31 ms, slice thickness of 3 mm, space thickness of 0.5 mm, and field of view (FOV) of 140 mm. The sequences of relevance for cartilage volume assessment were 3-dimensional sagittal water excitation dual echo steady state (DESS WE) images with time to recovery (TR) of 18.2 ms, time to echo (TE) of 5.28 ms, slice thickness of 1.3 mm, and field of view (FOV) of 140 mm.

### BML assessment

BMLs were evaluated on IW FS MR images at baseline and 2-years. We defined BMLs as ill-defined areas of high-signal intensity located within 1.0 cm of articular cartilage and present on either ≥ 2 sagittal images and/or ≥ 2 coronal images and classified them within 4 regions: index femoral region (representing the femoral region of the index compartment), non-index femoral region (representing the femoral region of the opposite compartment), index tibial region, and non-index tibial region. Prior to measuring BML size, image brightness and contrast were adjusted until normal bone signal appeared black and homogenous. Adjustments of image contrast and brightness, as well as BML identification, were conducted on paired images (baseline and 24 months). One reader (JD) measured the maximal anterior-posterior (AP), medial-lateral (ML), and superior-inferior dimension of each BML using the linear measurement tool in eFilm Workstation 3.01 (Merge Healthcare, Milwaukee, WI; Figure 1). AP and superior-inferior dimensions were measured on sagittal images and the ML dimension was determined on coronal images. For each lesion, the linear measurement tool was used to mark the BML edges that defined the greatest diameter for each dimension (e.g. AP, ML) on the sagittal and coronal images with the largest BML cross-section. The line measurements were then copied to all images within the image set and the reader reviewed the adjacent images to determine if the BML extended beyond the measurement lines in any of the other slices. If the BML extended beyond the line then the measurement line was lengthened to represent the maximum width of the BML across images.

We took the product of the three linear measurements to represent the approximate volume of each BML. When multiple BMLs were present in a region (e.g. index femur) their approximate volumes were summed to calculate regional approximate volume. Regional approximate BML volume changes were defined as progression if the volume increased and regression if the volume decreased. We also measured peak signal intensity in each BML on coronal images. Peak signal intensity was normalized to normal bone marrow signal to correct for variations in signal intensity that occurs across MR image acquisitions. Normal bone marrow signal intensity was collected from the posterior non-index femur because this region consistently had no BMLs. When multiple BMLs were present, the BML with greatest signal intensity defined the regional peak signal intensity. Intra-tester reliability (intraclass correlations [ICC 3,1 model])[15] for BML measurements ranged from 0.87 to 0.98 for BML linear measurements; 0.96 and 0.90 for volume and volume change; 0.88 for peak signal intensity. The manually-measured approximate BML volumes were correlated to segmentation BML volumes performed by an independent rater (JP; ICC [2,1] = 0.81, n = 17 BMLs). Furthermore, a Bland-Altman analysis indicated the 95% limit of agreement between manual and segmented BML size ranged from -2.8 to 1.3 cm^3^. Sixteen of the 17 manually-measured approximate BML volumes were in agreement with the segmented BML volume. Manual measurements were always greater than the segmented BML volumes (ranged from -3.8 to 0.0 cm^3^).

### Cartilage assessment

**Figure 2:**
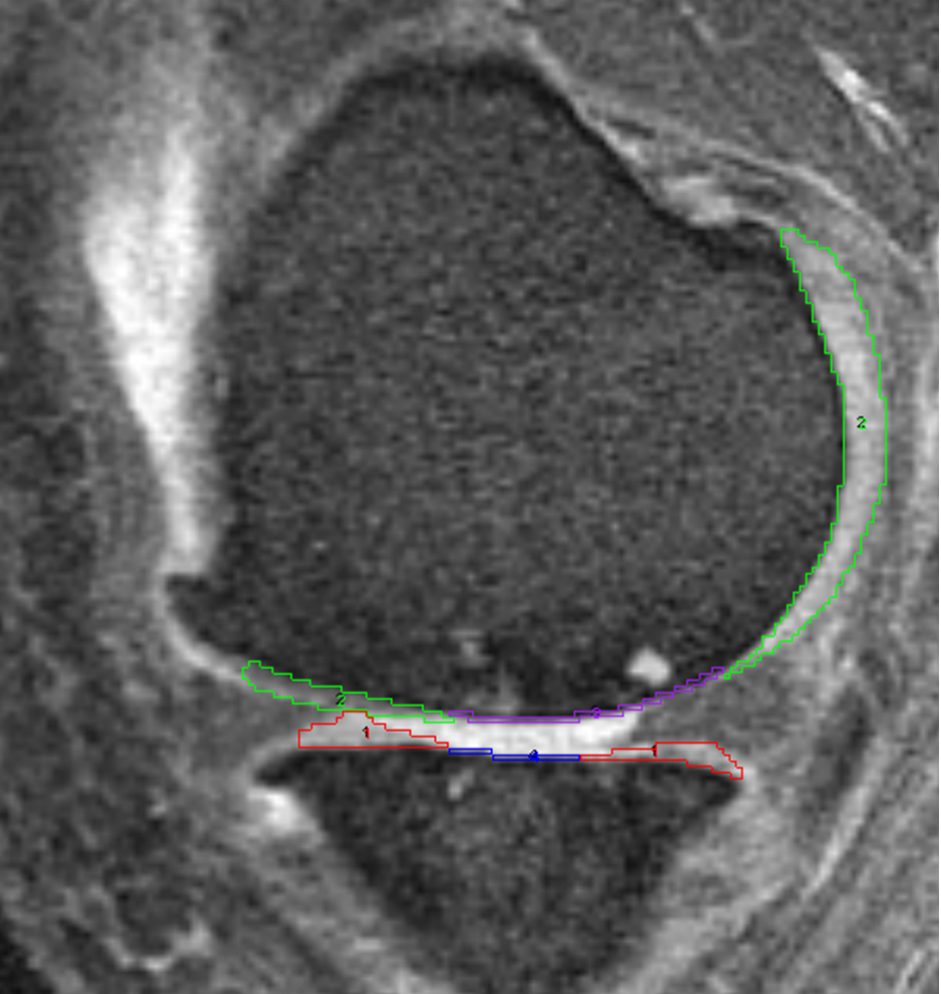
**Cartilage segmentation (green and red outlines) with lines (purple and blue) in areas of a full thickness cartilage lesion**.

Cartilage parameters were evaluated on sagittal DESS MR images at baseline and 24 months. Using Analyze 8.1^© ^(Mayo Clinic, Rochester, MN), one investigator (JYL), unblinded to the image acquisition order, registered the baseline and 24 month MR images and then performed paired manual segmentation of the index-compartment tibia and femur cartilage (Figure 2). Peripheral osteophytes were excluded in image segmentation. After completion of the manual segmentation cartilage volume (mm^3^) was calculated using Analyze (intra-tester ICC > 0.99).

To assess areas of full-thickness cartilage lesions, a second investigator (JBD) used Analyze 8.1^© ^to place a line in areas of full thickness cartilage lesions (Figure 2). In full thickness cartilage lesions with a central osteophyte the segmentation line was placed through the base of the osteophyte [16]. A MatLab (The MathWork, Natick, MA) custom program (written by EM) then analyzed these images and calculated mean cartilage thickness (with regions of full thickness cartilage lesions represented as 0 mm) as well as areas of full thickness cartilage lesions.

The custom MatLab program used the pixels identified as cartilage in each image. Binary morphological processing methods [17,18] were employed to determine the mean cartilage thickness. For each image slice the thickness of each connected component (CC) of the cartilage was computed as the mean distance from each pixel on the boundary of that CC from the morphological skeleton (a generalization of the centerline to arbitrary shapes) of that CC. This mean cartilage thickness was computed as twice the total distance to the skeleton of all points along the perimeter divided by the number of points times the linear size of each voxel.

To determine the length of full thickness cartilage lesions in each MR image slice the following process was undertaken. First, the morphological skeletons were computed for each pair of connected cartilage segments that was separated by a full thickness cartilage lesion. A second order polynomial was fit to the points from both morphological skeletons resulting in a curve that "spanned" the full thickness cartilage lesion between the two pieces of cartilage. The portion of this curve not overlapping the two cartilage pieces was taken to be the length of the full thickness cartilage lesion. To determine the mean cartilage thickness including the full thickness cartilage lesion, we estimated the number of pixels that comprise the full thickness cartilage lesion as the product of the full thickness cartilage lesion length and the density of pixels (pixels per unit length) as computed from the two bordering cartilage segments. The mean cartilage thickness including the full thickness cartilage lesion in each slice was then computed as previously described, but the number of points in the denominator was the sum of the number of pixels associated with both the full thickness cartilage lesion and cartilage segments. The mean cartilage thickness (with regions of full thickness cartilage lesions represented as 0 mm) was averaged across slices.

Finally, the area of full thickness cartilage lesion was computed as the ratio of the area of the full thickness cartilage lesion to the area of the full thickness cartilage lesion plus the surface area of the cartilage pieces (total area of subchondral bone) where the latter was calculated as the curve length of the second order polynomial that did intersect the two pieces of cartilage [17,18].

### Statistical analyses

Descriptive data were calculated for the study sample. Change in approximate BML volume was classified as regression (getting smaller), progression (getting larger), or no change in size relative to measurement error (test, retest). Longitudinal BML measurement error was calculated with the reliability data set (described above) by calculating the difference between the first approximate BML volume change and the retest approximate BML volume change (Differences in approximate BML volume change = [Follow-up_1 _- Baseline_1_] - [Follow-up_2 _- Baseline_2_]). Smallest detectable differences were originally calculated based on two standard deviations of the differences in approximate BML volume change (i.e., 95% confidence interval [95% CI] of the mean), however the data were not normally distributed and the sample size was small (*n *= 16 BMLs, mean = 1.86 cm^3^, median = 0.4 cm^3^, standard deviation = 5.99 cm^3^). Therefore, the smallest detectable difference was derived from the 95% CI of the median (median = 0.4 cm^3^; 95% CI = -0.1 to 3.2 cm^3^). To be conservative, we chose the larger endpoint of the 95% CI of the median. BML regression was defined as an approximate BML change less than -3.2 cm^3 ^over 2 years and BML progression was defined as an approximate BML change greater than 3.2 cm^3 ^over 2 years (changes between -3.2 cm^3 ^and 3.2 cm^3 ^were classified as no change). To further explore this definition of change we evaluated a boot-strapping resampling method (1000 resamples). This method and the 95% CI of the median resulted in a similar distribution for classifying BMLs as regression, no change, and progression.

Regional BML cross-sectional and longitudinal descriptive characteristics were limited to participants with BMLs present in the specific region (e.g. index femur). We used Spearman correlations to evaluate the intra-regional associations between regional baseline and longitudinal BML volume as well as regional approximate BML volume and extent of cartilage damage. These correlations were limited to a sample of knees with full thickness cartilage lesions at baseline (see the Study Sample section for justification). Fisher's z transformations were performed to determine 95% CI for correlation coefficients. To assess the external validity of the correlations between approximate BML volume and extent of cartilage damage, Spearman correlations were analyzed, using the entire cohort (*n *= 107), to evaluate the intra-regional associations between regional approximate BML volume, cartilage volume, and cartilage thickness. Differences in correlation coefficients between the entire cohort and subset with full thickness cartilage lesions were defined by coefficients being within the 95% confidence intervals of the correlation coefficient that it was being compared. Bonferroni corrections were used to correct for the 24 Spearman correlations that evaluated the hypotheses that approximate BML volumes were associated with cartilage damage (adjusted p value for significance was p < 0.002). Significant univariate correlations were explored with follow-up stepwise multiple linear regression models to determine if potential covariates (i.e., age, sex, body mass index, intervention group) influenced the associations.

Within the index compartment structural changes in the femur or tibia may alter the loading throughout the compartment. Therefore, exploratory Spearman correlations evaluated the inter-regional relationships within the index compartment between regional approximate BML volume and extent of cartilage damage. We used SAS 9.2 (SAS Institute, Cary, NC) to calculate regional approximate BML volumes, derive median and 95% CI for the median as well as perform all statistical analyses.

## Results

### Descriptive characteristics of regional approximate BML volume and signal intensity

**Figure 3:**
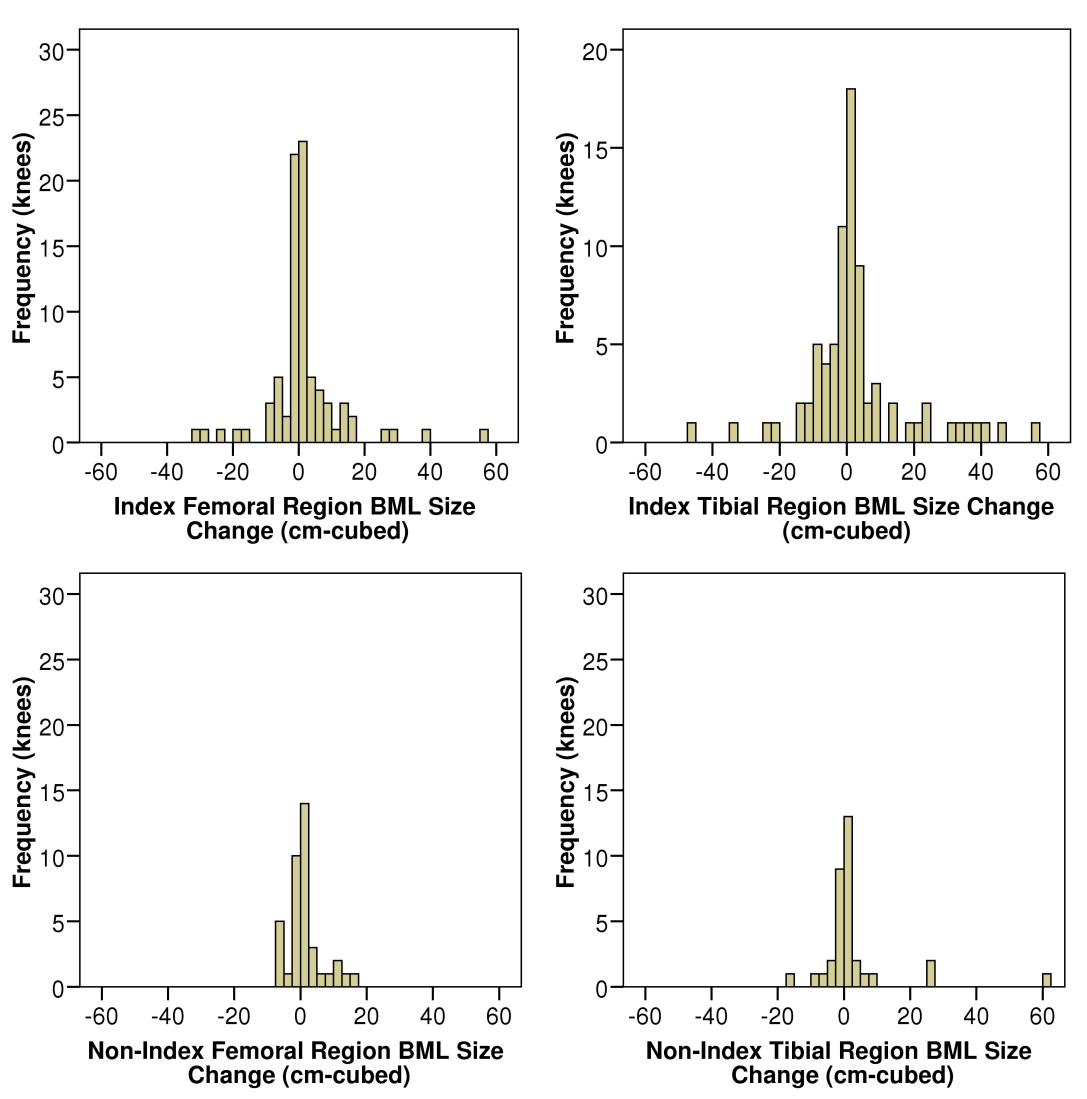
**Histograms of bone marrow lesion (BML) size change among regions with BMLs**. Each bar represents a range of 2.5 cm^3^.

From the original trial, 107 participants were included in the analyses with a mean age of 63 ± 9 years, mean body mass index of 29.8 ± 5.4 kg/m^2^, 64% female; and 52% KL grade 2 (56 knees),30% KL grade 3 (32 knees), and 18% KL 4 grade (19 knees). In this sample 101 (94%) knees had ≥ 1 BML (range 1 to 7) for a total of 240 BMLs. Furthermore, 45 (19%) BMLs regressed (got smaller), 61 (25%) BMLs progressed (got larger), and 134 (56%) did not change over two years. Table 1 provides regional baseline approximate BML volumes. While most regions demonstrated no or small regional approximate BML volume changes over 2 years, some underwent large changes (Figure 3, Table 1). Longitudinal changes in regional approximate BML volume were not related to baseline approximate BML volumes (Table 1; see scatter plots in Additional File 1).

**Table 1:** Median (95% CI) Baseline and Longitudinal Changes in Approximate BML Volume

Region	Baseline BML Volume (cm^3^)	BML Volume Change (cm^3^)	Spearman Correlation (95% CI): Baseline to Longitudinal Change
Index Femur (*n *= 76)	4.2 (2.5 to 11.5)	0.1 (-0.5 to 0.9)	-0.20 (-0.41 to 0.02)
Index Tibia (*n *= 76)	8.8 (3.9 to 14.3)	0.5 (-0.3 to 1.7)	-0.14 (-0.35 to 0.09)
Non-index Femur (*n *= 33)	2.6 (1.6 to 8.2)	0.4 (-0.2 to 1.6)	-0.06 (-0.40 to 0.29)
Non-index Tibia (*n *= 30)	1.1 (0.6 to 3.9)	0.2 (-0.1 to 1.2)	-0.27 (-0.57 to 0.10)

No Spearman correlation coefficients were statistically significant. 95% CI = 95% confidence interval of the median or 95% confidence interval of the Spearman Correlation. See Additional File 1 for scatter plots of the analyzed correlations.

### Relationships between approximate BML volume and cartilage parameters within index compartments

To compare the cross-sectional and longitudinal relationships between regional approximate BML volume and the extent of hyaline cartilage damage, a sample consisting of 44 knees was selected (65 ± 9 years of age (mean ± standard deviation), 52% female, and body mass index of 29.8 ± 5.8 kg/m^2^). Among index femurs, 91% had a full thickness cartilage lesion at baseline (baseline cartilage lesion = 12.1 ± 10.3% of subchondral bone; 2 knees developed a full thickness cartilage lesion when none was present at baseline; change = 1.5 ± 1.8% of subchondral bone) and 91% had baseline BMLs (baseline BML size = 15.3 ± 16.0 cm^3^; 1 knee developed a BML when none was present at baseline; 4 knees had a BML at baseline that was absent at follow-up; change = 1.6 ± 12.4 cm^3^). Among index tibiae, 66% had a full thickness cartilage lesion at baseline (baseline cartilage lesion = 24.8 ± 17.8% of subchondral bone; 3 knees developed a full thickness cartilage lesion when none was present at baseline; change = 3.0 ± 4.7% of subchondral bone) and 84% had baseline BMLs (baseline BML size = 30.7 ± 31.5 cm^3^; 2 knees developed a BML when none was present at baseline; change = 3.9 ± 14.8 cm^3^).

Table 2 describes the intra-regional correlations, in the index compartment, between cartilage and regional approximate BML volume (see scatter plots in Additional File 1). After Bonferroni adjustments for multiple testing only two statistically significant correlations were present between regional approximate BML volume and cartilage parameters: baseline tibia BML volume to baseline tibia full thickness cartilage lesion area and baseline femur BML volume to change in femoral full thickness cartilage lesion area. There were no other statistically significant correlations between cartilage parameters (baseline or change) and BML volume change. To assess the external validity of these findings, approximate BML volume and extent of cartilage damage correlations between approximate BML volume, cartilage volume, and cartilage thickness were evaluated and determined to be similar between the subset (*n *= 44) and entire cohort (*n *= 107; see table in Additional File 2). Based on stepwise multiple linear regressions age, sex, body mass index, or intervention group did not influence significant univariate associations.

**Table 2:** Within-Region Spearman Coefficients (95% Confidence Intervals) Among Baseline BML and Cartilage Parameters in the Index Compartment

	Femur BML Volume: Baseline (n = 40)	Tibia BML Volume: Baseline (n = 37)	Femur BML Volume: Change (n = 41)	Tibia BML Volume: Change (n = 39)
Baseline				
Cartilage Volume	-0.06 (-0.37, 0.25)	-0.31 (-0.57, 0.02)	-0.06 (-0.37, 0.25)	-0.08 (-0.39, 0.24)
Cartilage Thickness	0.02 (-0.30, 0.33)	-0.37 (-0.62, -0.05)	-0.12 (-0.42, 0.19)	-0.02 (-0.33, 0.30)
Full Thickness Cartilage Lesion Area	0.33 (0.02, 0.58)	0.63 (0.38, 0.79)*	-0.06 (-0.36, 0.26)	-0.03 (-0.34, 0.29)
2-year Longitudinal Change				
Cartilage Volume	0.03 (-0.29, 0.34)	0.16 (-0.18, 0.46)	-0.10 (-0.39, 0.22)	0.32 (0.00, 0.58)
Cartilage Thickness	-0.11 (-0.41, 0.21)	-0.13 (-0.43, 0.21)	-0.15 (-0.43, 0.17)	0.04 (-0.28, 0.35)
Full Thickness Cartilage Lesion Area	0.48 (0.20, 0.69)*	0.43 (0.12, 0.66)	0.08 (-0.23, 0.38)	-0.18 (-0.47, 0.14)

* statistically significant finding after Bonferroni corrections (24 multiple comparisons; p < .002; see Additional File 1 for scatter plots).

Exploratory correlations between index-compartment regions were examined: index-femoral baseline BML with index-tibial baseline full thickness cartilage lesion area (*r *= 0.40, *p *= 0.01; *n *= 40), index-femoral baseline BML with index-tibial full thickness cartilage lesion area change (*r *= 0.39, *p *= 0.01; *n *= 40), index-tibial baseline BML with index-femoral baseline full thickness cartilage lesion area (*r *= 0.55, *p *< 0.001, *n *= 37), index-tibial BML change with index-femoral baseline cartilage volume (*r *= -0.33, *p *= 0.04, *n *= 39), and index-tibial BML change with index-femoral baseline cartilage thickness (*r *= -0.38, *p *= 0.02, *n *= 39). No other statistically significant correlations were detected across regions of the index.

## Discussion

This study used a simple and novel method of measuring approximate BML volume that can be easily employed by researchers and clinicians. The technique demonstrated that many knee regions experienced small longitudinal changes in regional approximate BML volume over 2 years (± 2.5 cm^3^) but some knees experienced large longitudinal changes. In agreement with previous findings, baseline regional approximate BML volume and hyaline cartilage full-thickness lesion area (baseline and longitudinal change) were associated [2,4,5,7,8,12,19-28]. The new BML measurement technique is sensitive to small changes in BML size over 2 years and, in agreement with previous literature, positively associated with increased cartilage damage.

**Figure 4:**
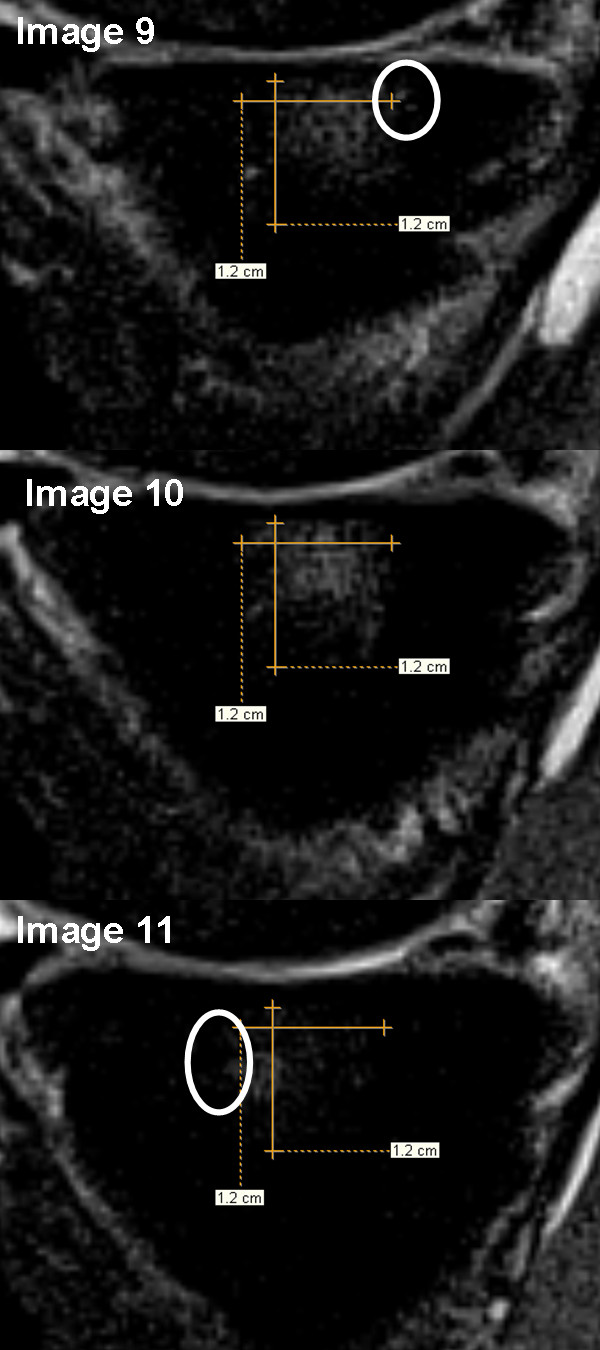
**Measurements of approximate BML volume based on only the coronal and sagittal images with the largest area of signal abnormality may misrepresent the greatest diameter of a BML**. Image 10, image with the largest BML area, would traditionally be measured to approximate BML volume and result in an anterior-posterior diameter of 1.2 cm but with the newer technique, measuring across images (including images 9 and 11), the anterior-posterior diameter is 1.5 cm. The white circles indicate areas of increased signal intensity beyond the dimensions indicated on image 10. The image contrast is optimized for measuring bone marrow lesions based on the measurement protocol (not for viewing other anatomical structures).

To our knowledge, this is the first full-length publication reporting the longitudinal relationship between 3-dimensional quantitative measurements of BML size and hyaline cartilage among knees with OA. Several methods have been deployed to quantify BML size but they have various limitations. One method uses manual segmentation of all MR image slices that display a BML. This method is time consuming and usually requires proprietary software [29-36]. Therefore, a segmentation technique is difficult to deploy researchers working with large cohorts. A less labor-intensive method that has been used is to take the measurement of the greatest diameter of a lesion. One potential downside to this approach is that it does not account for the three-dimensional nature of these lesions [7]. An alternative method that is not very labor intensive approximates BML volume with three linear measurements; specifically, the coronal and sagittal images with the largest area of signal abnormality are identified and the largest three dimensions from these two images [37,38]. While this appears to be a good compromise between function and practicality, this method does not take into account for the possibility that the greatest width of a BML may change as you view adjacent coronal or sagittal images (Figure 4). By measuring the greatest diameter across images the current technique attempts to account for the potential limitations that a single image may not adequately describe the 3-dimensional size of an irregularly shaped BML.

Baseline regional approximate BML volume is positively associated with baseline and longitudinal changes in full thickness cartilage lesion area. A positive correlation between semiquantitative assessments of BML size and cartilage damage have been demonstrated in cross-sectional [4,20-24] and longitudinal studies [2,4,5,8,19,24,25]. Previous reports regarding the within-region association between BML size and cartilage volume, the most common quantitative measure of hyaline cartilage, are conflicting with some showing statistically significant associations [7,12,21,24,26-28] and others showing non-significant associations [2,7,20,21,25,27]. The current correlations concur with previously reported associations between longitudinal change of BML size (greatest diameter) and cartilage volume (*r *= -0.03 to -0.40)[7]. Furthermore, one article, which distinguished between full thickness cartilage lesions and normal or partial thickness lesions, noted that only 7% of knees (11/149 knees) with normal cartilage or partial thickness defect had a BML greater than 1 cm in size while 33% of knees (16/48 knees) with a full thickness defect had a BML greater than 1 cm [10]. These data support the current findings that BML size may have a strong association with full thickness cartilage lesion area.

Baseline regional approximate BML volume was statistically associated with baseline and longitudinal changes in full thickness cartilage lesion area but not cartilage thickness or volume. The similar findings between cartilage thickness and volume are not unexpected since the parameters are based on similar cartilage data (e.g. cartilage thickness determines cartilage volume). Although the design of this study did not allow us to determine why regional BML volumes had stronger correlations with full thickness cartilage lesion area compared to cartilage volume or thickness, we speculate that BMLs are an early and sensitive biomarker of localized degeneration in response to altered loading. Furthermore, BMLs may also contribute to localized cartilage loss leading to full thickness cartilage lesions. A second hypothesis is that BMLs have a localized influence on cartilage loss and full thickness cartilage lesion area is a more localized measure of hyaline cartilage loss than the other two measures. These hypotheses are supported by the associations between baseline BML size and longitudinal changes in full thickness cartilage lesion area. A third hypothesis is that the association between BML size and cartilage volume and thickness is impeded because OA progression defined by cartilage thickness and volume is not always linear. Some patients experience increased cartilage thickness with KL grade 2 (23% of knees) and KL grade 3 (18%) while the majority lose cartilage thickness and volume [39]. In contrast, full thickness cartilage lesion area progresses in a linear pattern, which supports its higher correlations with BML size.

Exploratory analyses across regions demonstrated statistically significant associations including relations between baseline cartilage and regional approximate BML volume change. When assessing predictors of longitudinal cartilage loss or BML volume change it is important to consider other regions. Altered structure in one region of the index compartment is likely to alter the loading in both index regions. Inter-regional associations between BML and cartilage parameters warrant its own research. Few studies have evaluated inter-region relationships between BMLs and cartilage parameters but there is previous research to support these relationships. Statistically significant inter-regional correlations between baseline BML size (semi-quantitative) and cartilage volume change have been reported [28]. When accounting for variables that predict disease progression (e.g. cartilage loss, BML progression) it may be important to consider the influence of other regions throughout the joint.

Our study showed that small changes in BML size were common but that there is a large variation among knees with OA. This corroborates findings from other investigators who also found that among OA knees with BMLs, there was a large variability of longitudinal BML size change [7,36]. In a small sample (*n *= 14), investigators using semi-automated BML segmentation showed that 43% increased BML volume (change greater than 5%), 36% decreased BML volume, and 21% did not change (± 5% change)[36]. Small longitudinal changes with large variability has also been reported with assessments of maximum BML diameter (i.e. coefficient of variation [CV] = 719%, 4,360%)[7].

While the findings of the current study are interesting, there are several limitations. Approximate BML volume over-estimates the true BML volume but may be strongly correlated to the true BML volume. Furthermore, the method may not detect subtle changes within the baseline approximate volume.

Our data support the construct validity of the new methodology. This technique is intended to be a simple and efficient method of measuring 3-dimensional BML size that can be done with standard MR imaging viewing software. Future research should determine the relationship between true BML volume determined through BML segmentation and approximate BML volume using this technique.

## Conclusions

In summary, this study demonstrates a method of approximating BML volume that can be readily adopted by clinicians and researchers. The measurements detected longitudinal changes during a two-year clinical trial. Furthermore, baseline BML size was associated to the longitudinal changes in full thickness cartilage lesion area. More research is warranted to investigate the longitudinal relationship between quantitative measures of tissue degeneration associated with OA.

## Competing interests

The authors declare that they have no competing interests.

## Authors' contributions

JBD contributed to the conception and design, collection and assembly of data, analysis and interpretation of data, drafting/revisions of article, as well as final approval of the article.

GL contributed to the analysis and interpretation of data, drafting/revisions of article, as well as final approval of the article. JYL contributed to the collection and assembly of data, revisions of article, as well as final approval of the article. RW contributed to the conception and design, revisions of article, as well as final approval of the article. EM contributed to the collection and assembly of data, analysis and interpretation of data, revisions of article, as well as final approval of the article. JP contributed to the collection and assembly of data, revisions of article, as well as final approval of the article. LLP contributed to the conception and design, assembly of data, analysis and interpretation of data, drafting/revisions of article, as well as final approval of the article. TEM contributed to the conception and design, analysis and interpretation of data, obtaining funding, drafting/revisions of article, as well as final approval of the article.

## Pre-publication history

The pre-publication history for this paper can be accessed here:

http://www.biomedcentral.com/1471-2474/12/217/prepub

## Supplementary Material

Additional File 1 **Intra-regional Scatter Plots**. Scatter plots for correlations presented in Table 1 and Table 2 are provided. Click here for file

Additional File 2 **Additional Table. Within-Region Spearman Coefficients (95% Confidence Intervals) Among Baseline BML and Cartilage Parameters in the Index Compartment**. This table demonstrates the correlations between approximate BML volume, cartilage volume, and cartilage thickness among the entire study cohort (*n *= 107). These correlations were evaluated and determined to be similar between the primary subset (*n *= 44; Table 2) and entire cohort (*n *= 107). Click here for file
