# Supplementary material for: Quantitative bone marrow lesion size in osteoarthritic knees correlates with cartilage damage and predicts longitudinal cartilage loss
Source: BMC Musculoskelet Disord. 2011 Sep 30;12:217. doi: 10.1186/1471-2474-12-217 (PMC3190349; doi:10.1186/1471-2474-12-217)

Intra-Regional Scatter Plots:

Baseline BML size to Longitudinal BML Size Change (Table 1)

Index Femur: Baseline BML size to Longitudinal BML Change

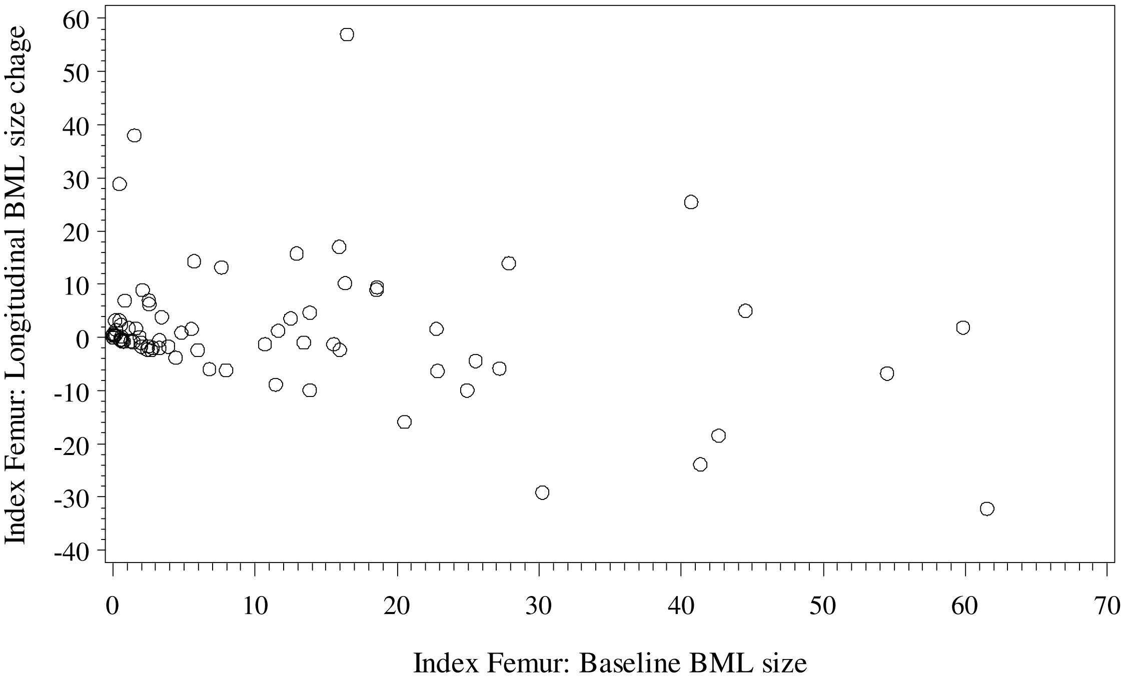

Index Tibia: Baseline BML size to Longitudinal BML Change

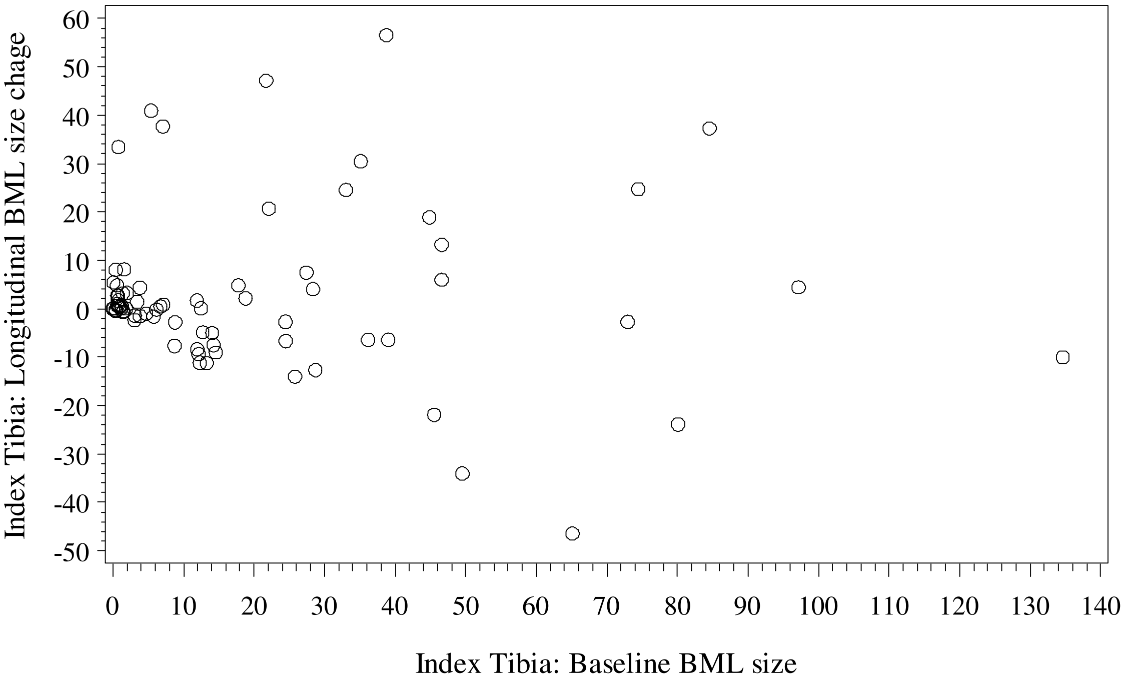

Non-index Femur: Baseline BML size to Longitudinal BML Change

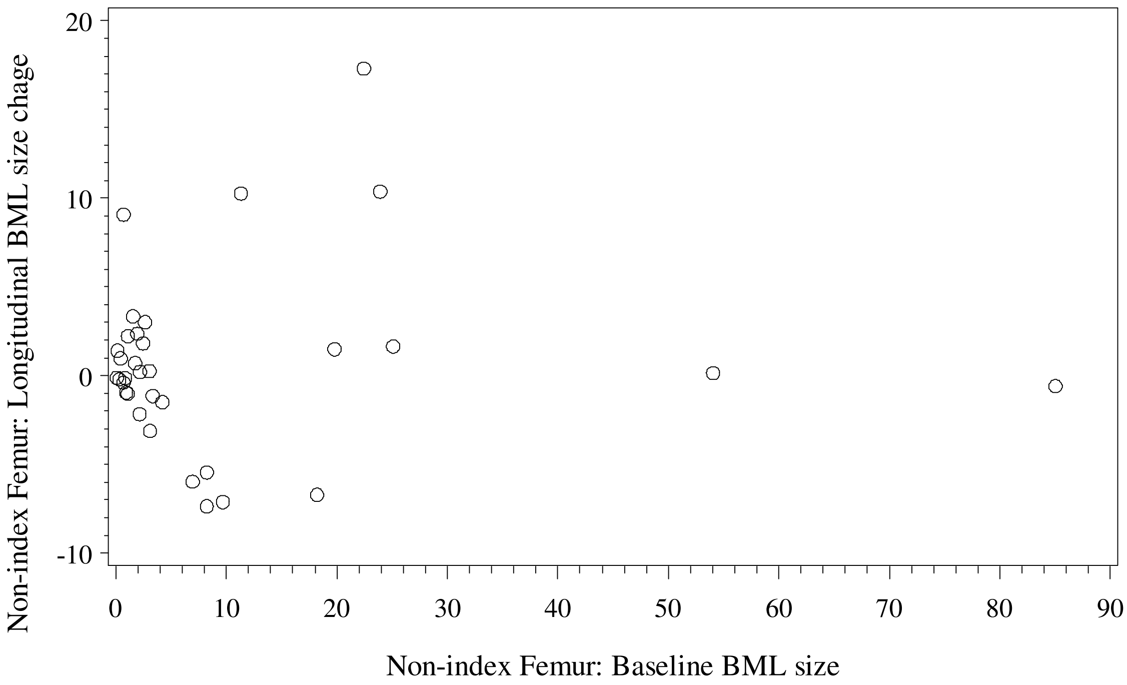

Non-index Tibia: Baseline BML size to Longitudinal BML Change

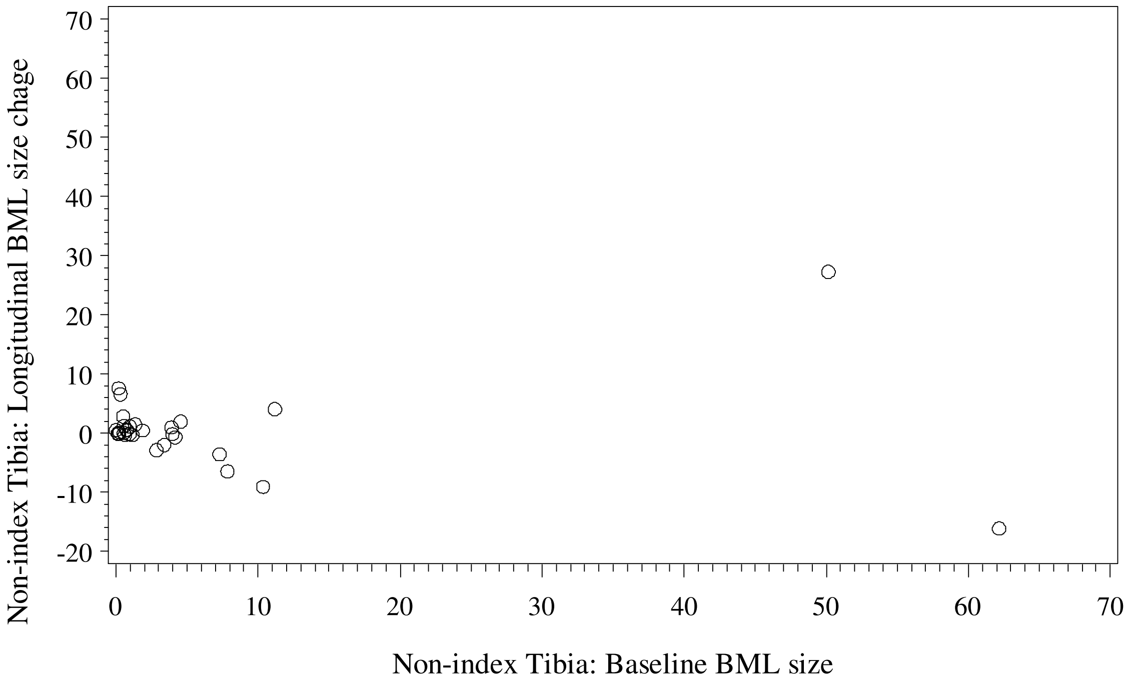

Approximate BML Volume and Cartilage Parameters (Table 2)

Index Femur: Baseline BML size to Baseline Cartilage Volume

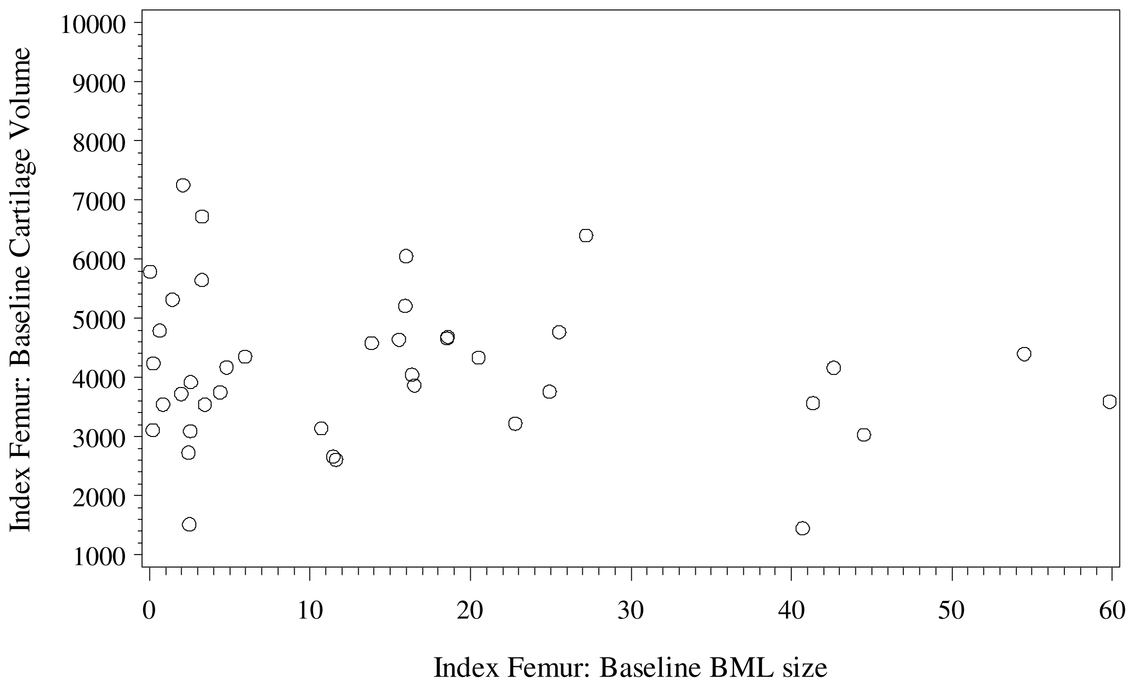

Index Femur: Baseline BML size to Baseline Cartilage Thickness

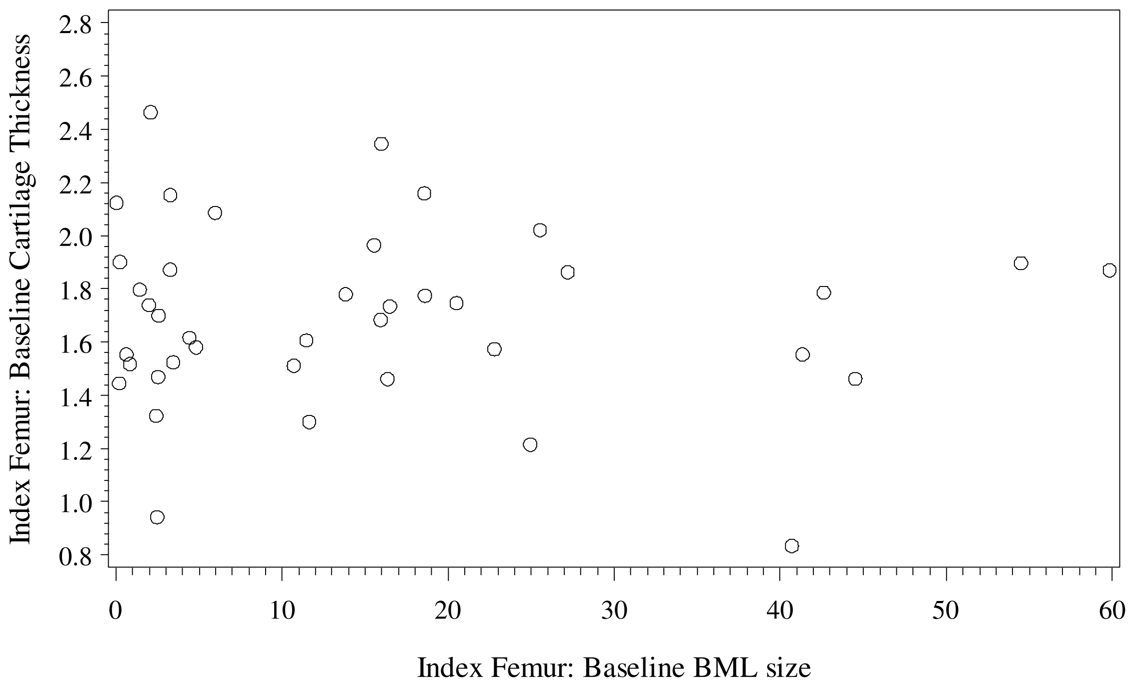

Index Femur: Baseline BML size to Baseline Full Thickness Cartilage Lesion Area

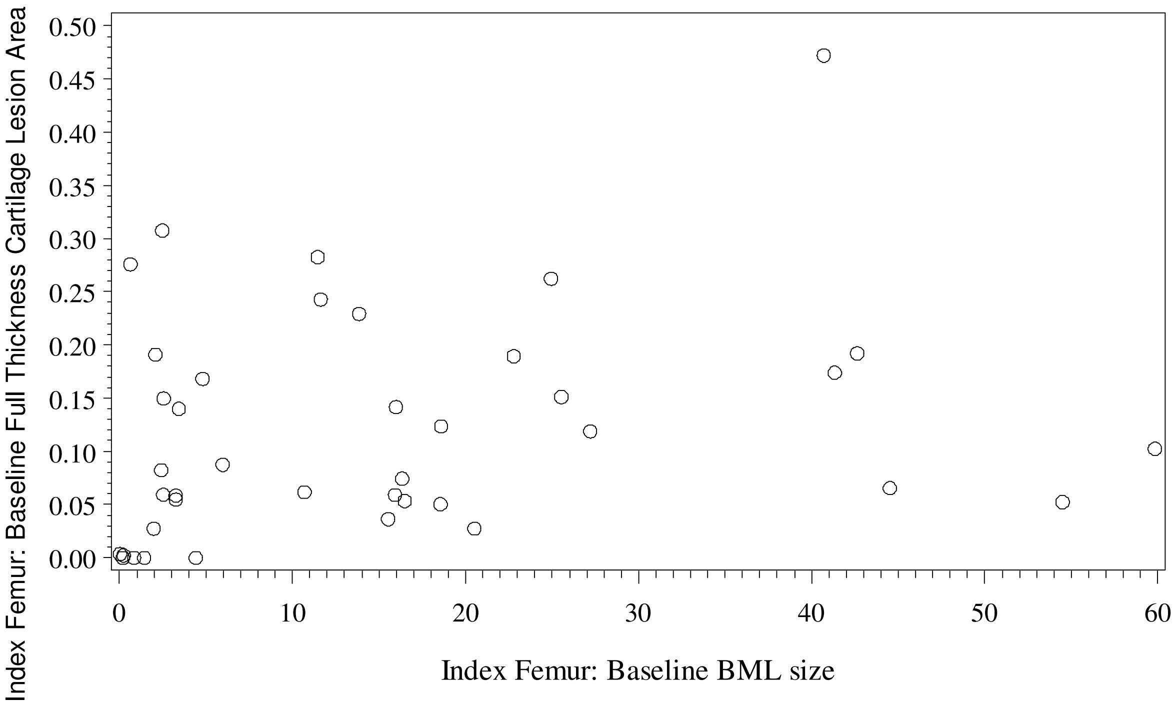

Index Femur: Baseline BML size to Longitudinal Cartilage Volume

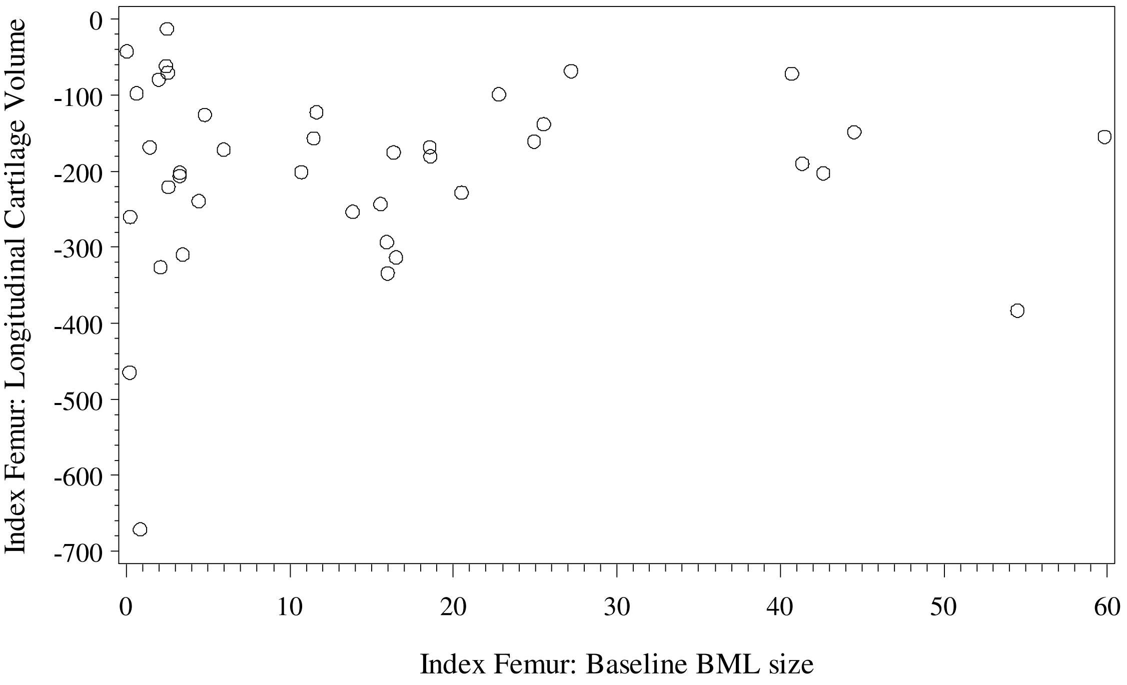

Index Femur: Baseline BML size to Longitudinal Cartilage Thickness

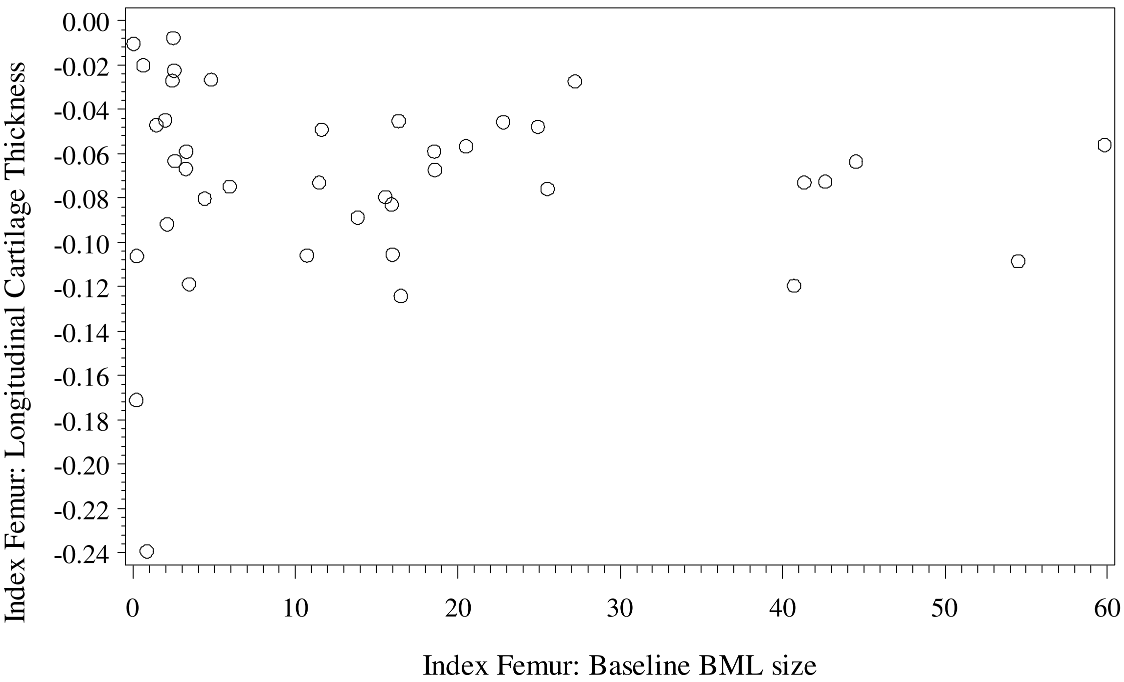

Index Femur: Baseline BML size to Longitudinal Full Thickness Cartilage Lesion Area

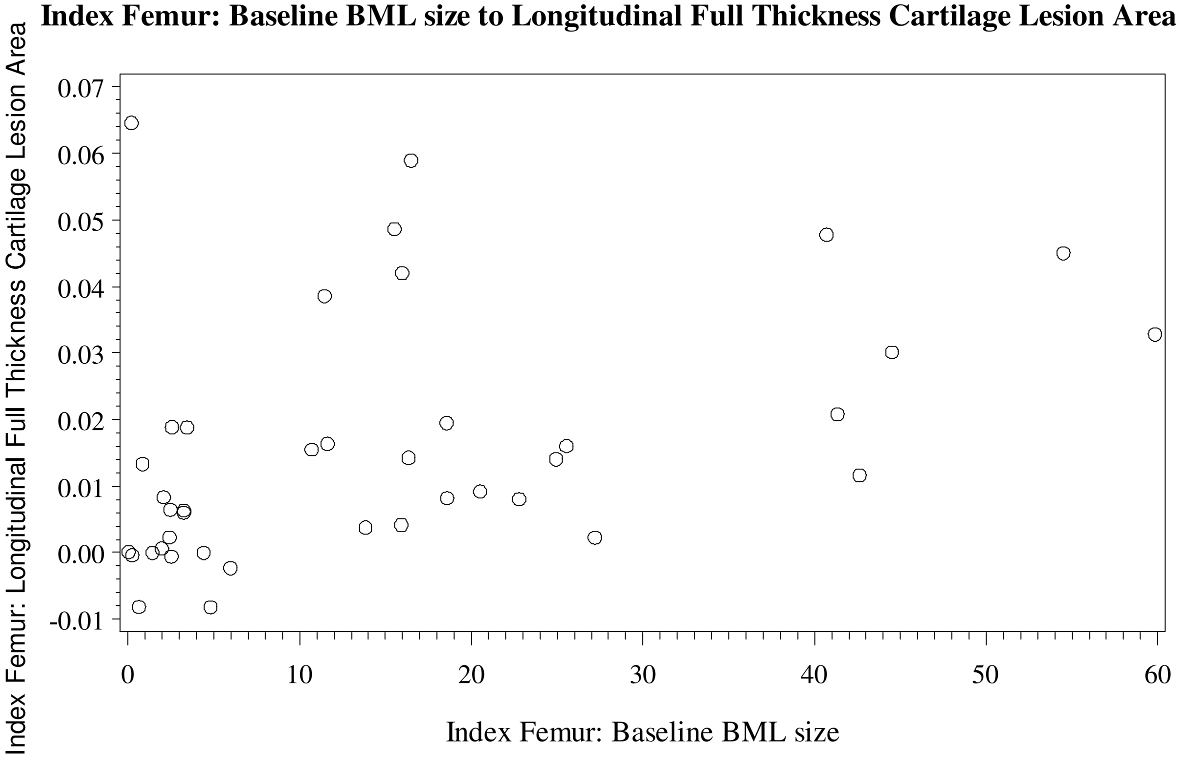

Index Tibia: Baseline BML size to Baseline Cartilage Volume

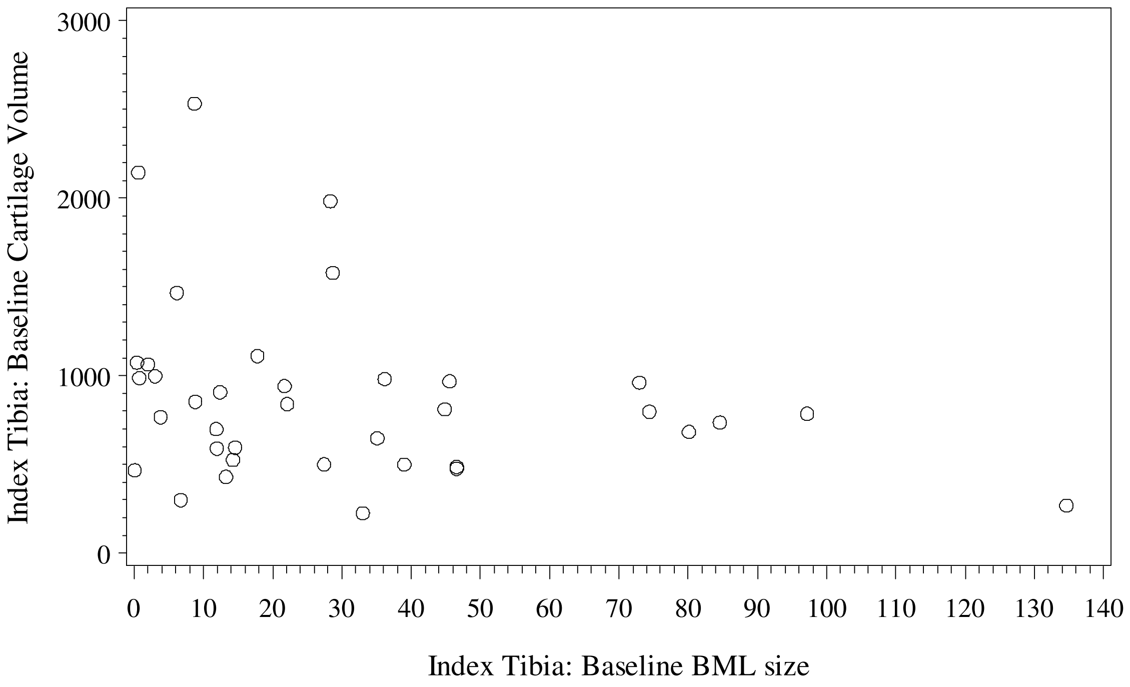

Index Tibia: Baseline BML size to Baseline Cartilage Thickness

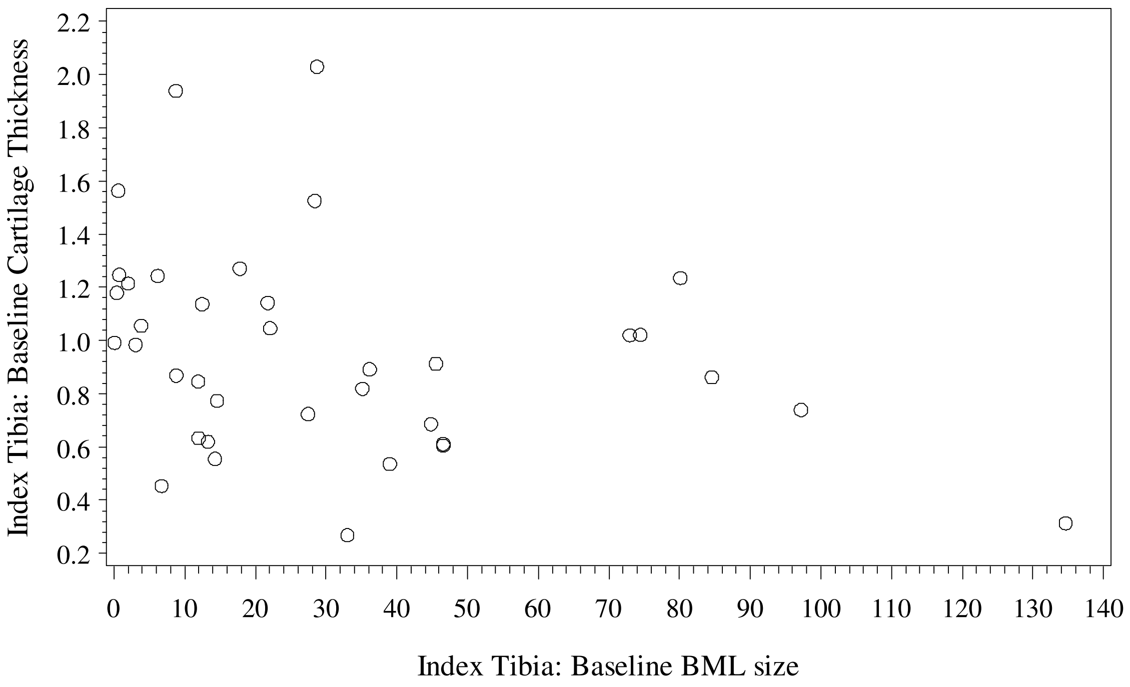

Index Tibia: Baseline BML size to Baseline Full Thickness Cartilage Lesion Area

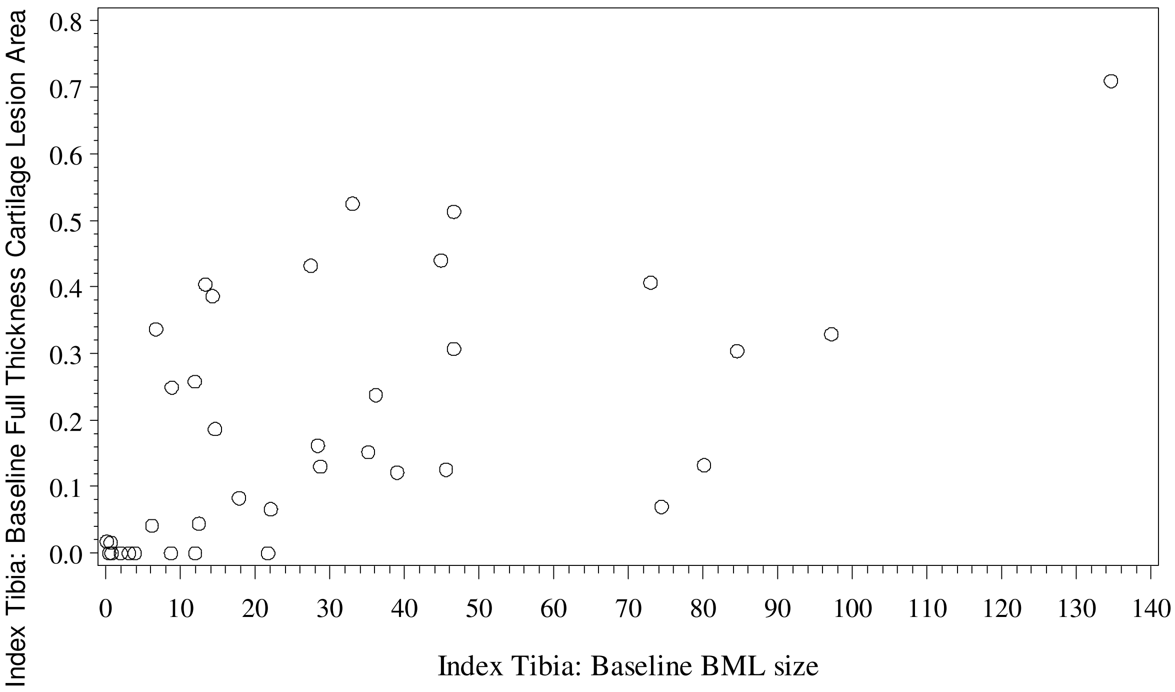

Index Tibia: Baseline BML size to Longitudinal Cartilage Volume

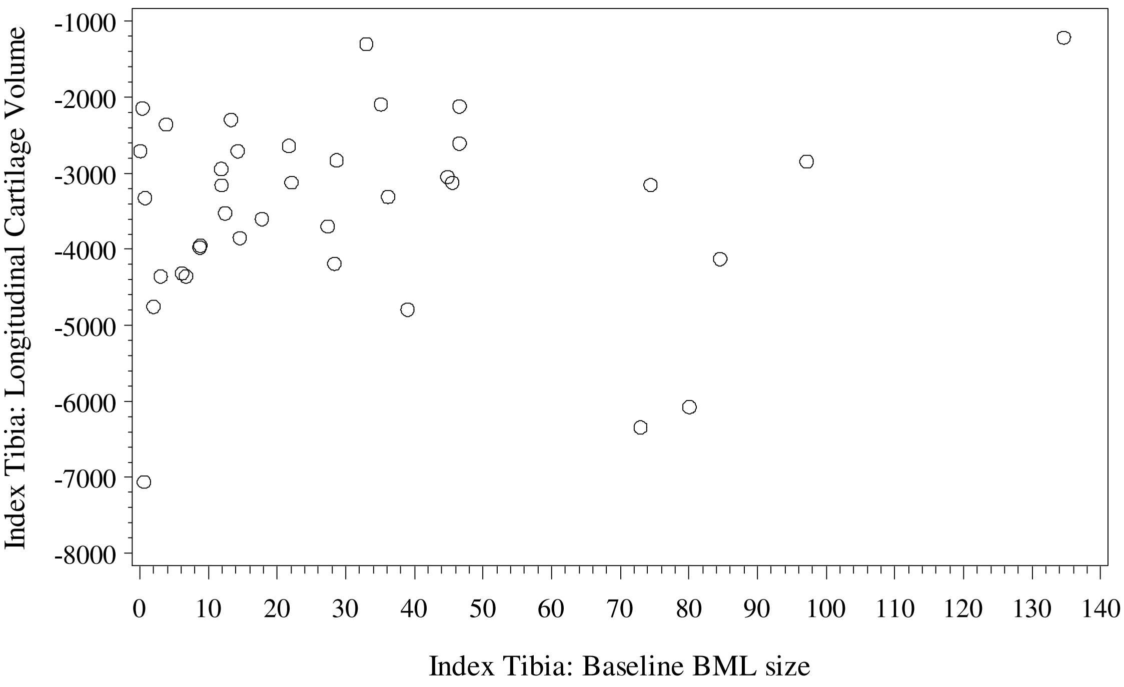

Index Tibia: Baseline BML size to Longitudinal Cartilage Thickness

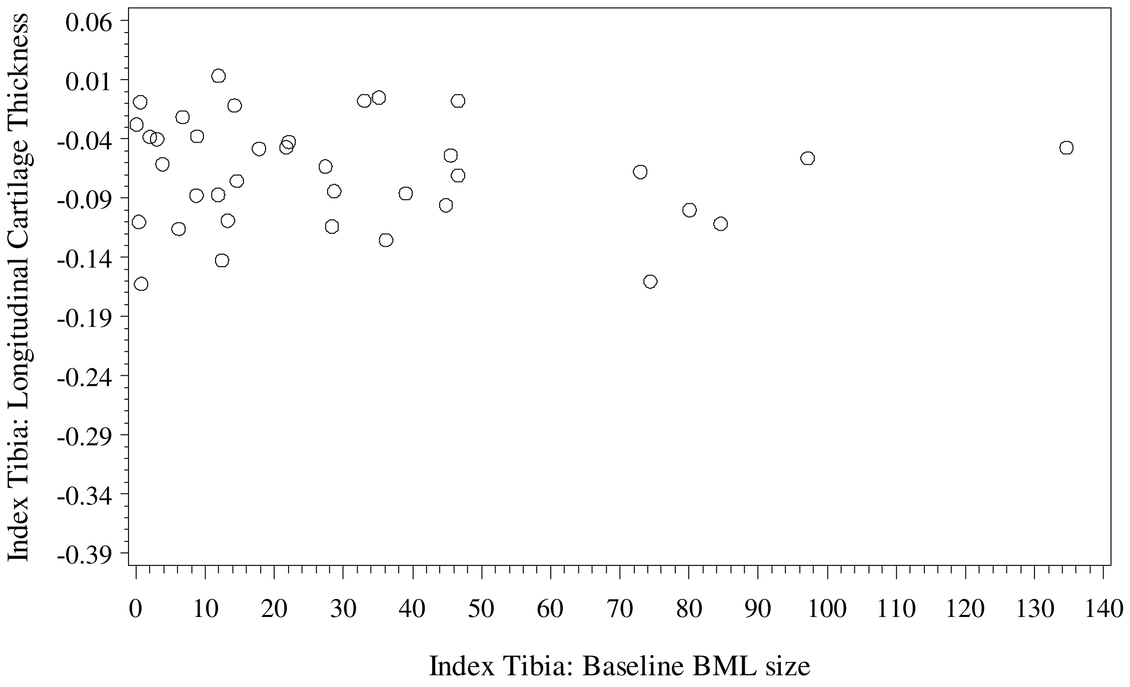

Index Tibia: Baseline BML size to Longitudinal Full Thickness Cartilage Lesion Area

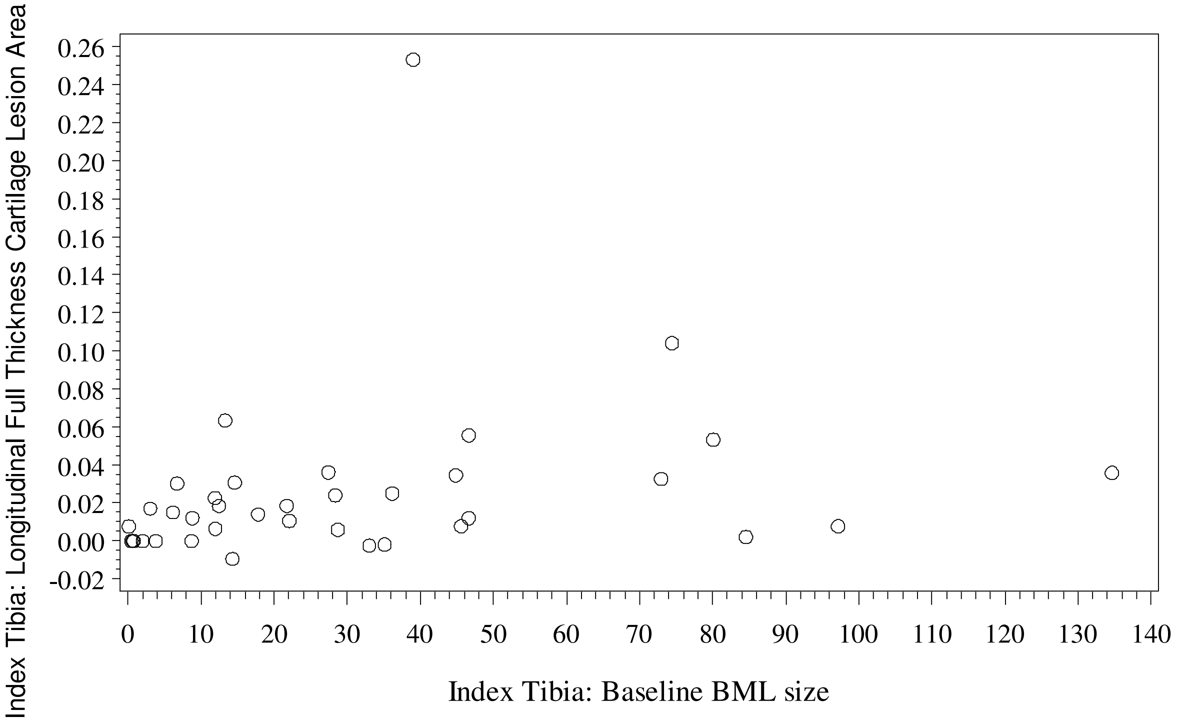

Index Femur: Longitudinal BML size to Baseline Cartilage Volume

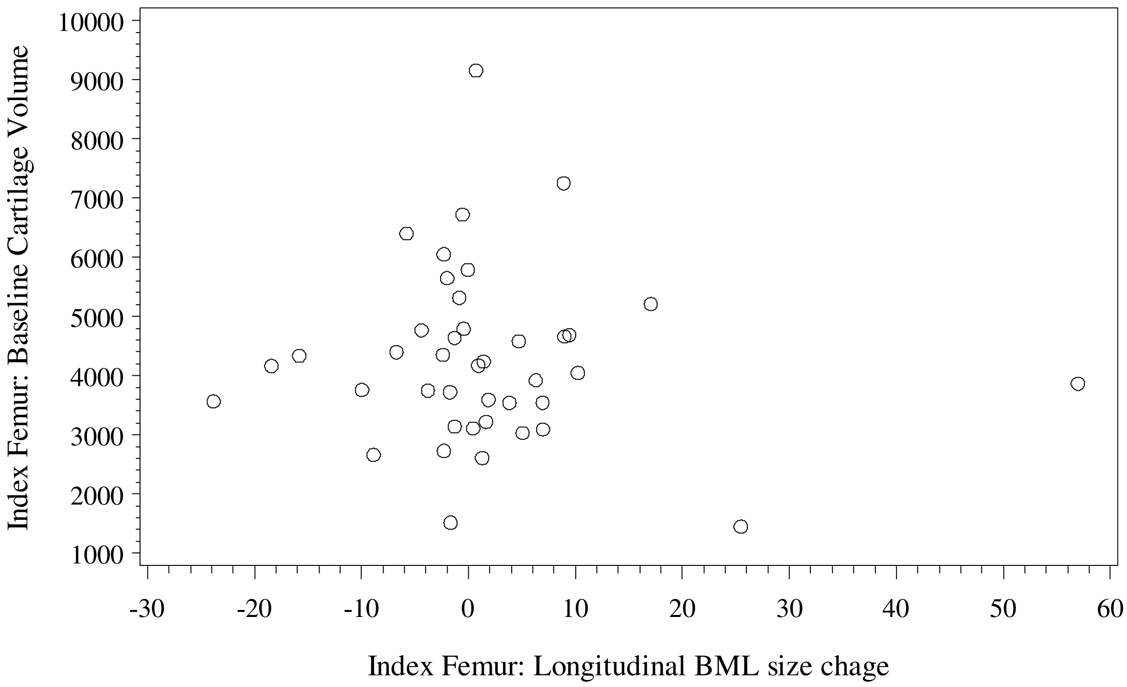

Index Femur: Longitudinal BML size to Baseline Cartilage Thickness

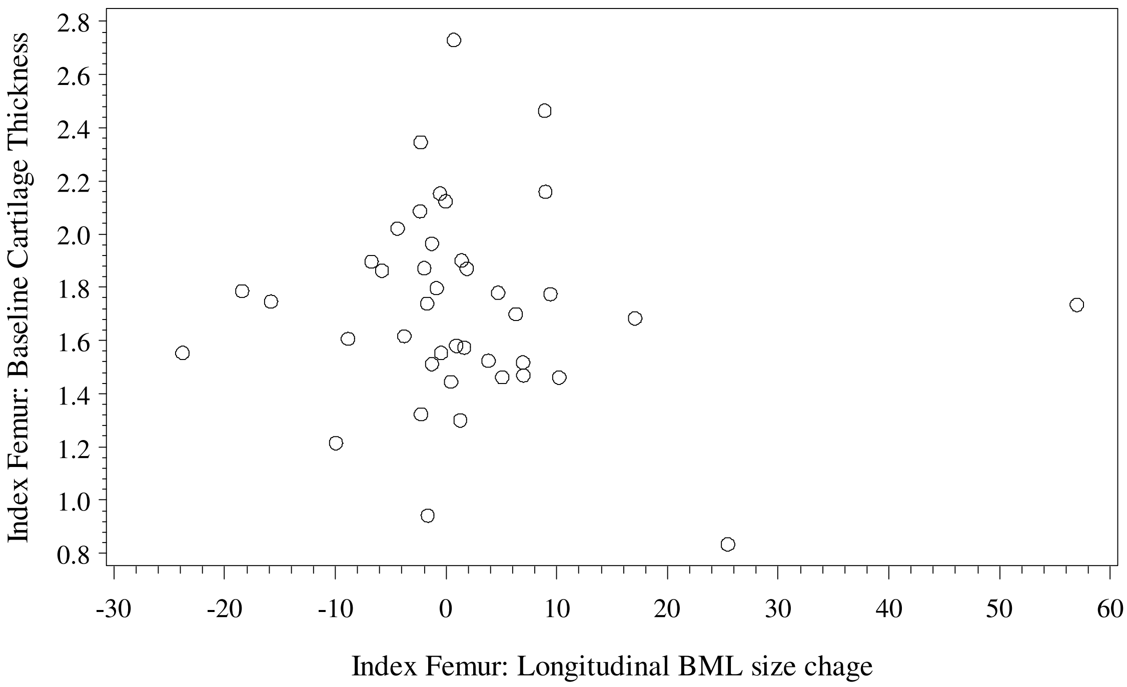

Index Femur: Longitudinal BML size to Baseline Full Thickness Cartilage Lesion Area

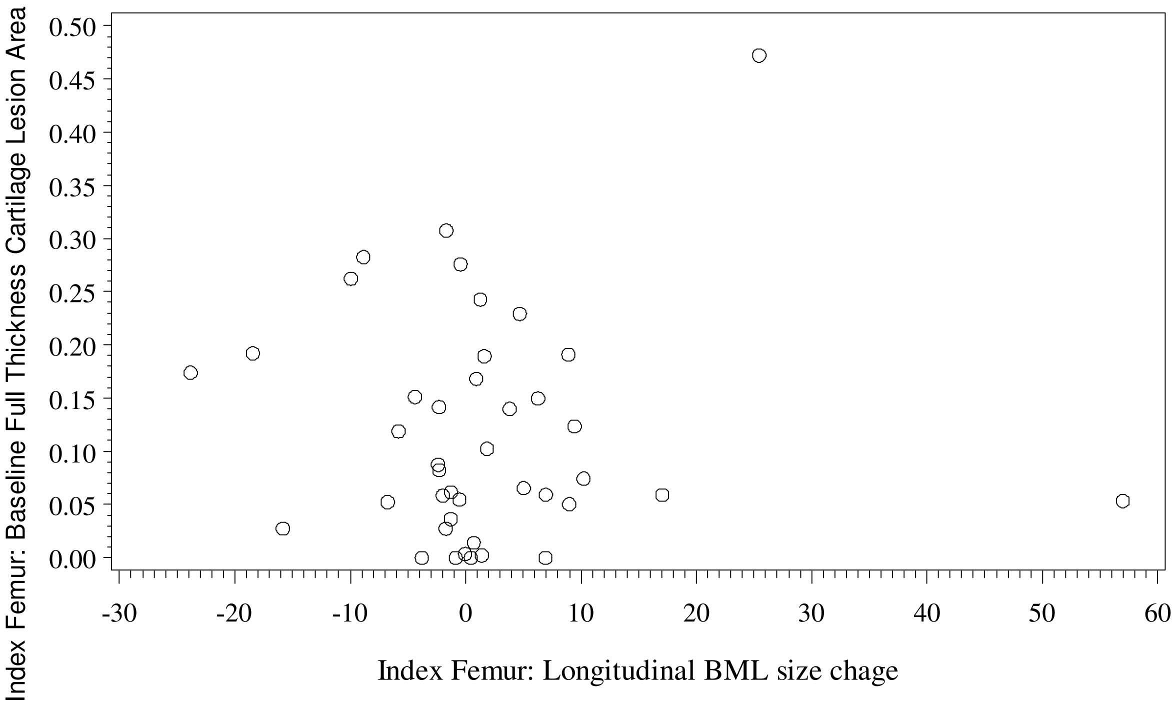

Index Femur: Longitudinal BML size to Longitudinal Cartilage Volume

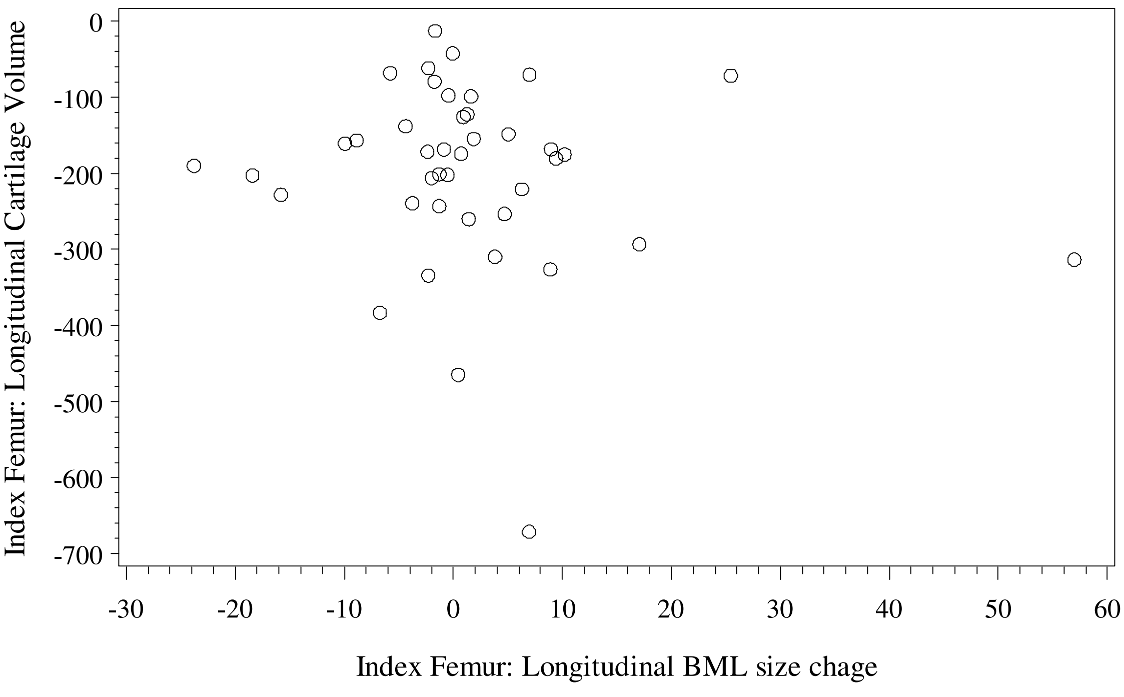

Index Femur: Longitudinal BML size to Longitudinal Cartilage Thickness

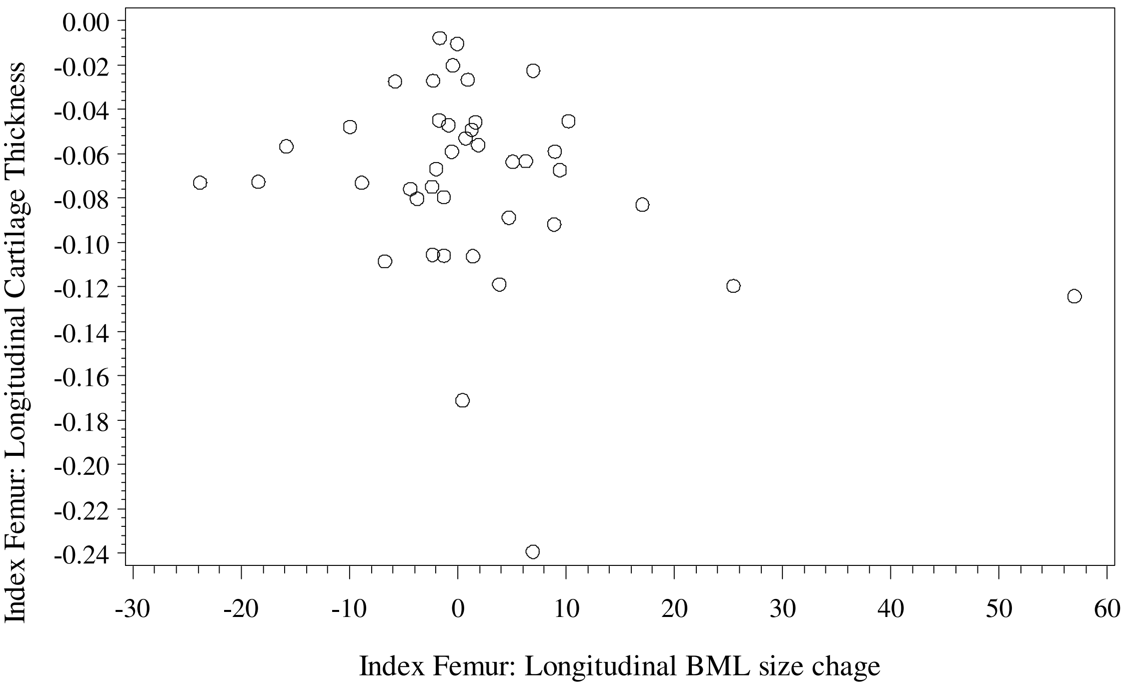

Index Femur: Longitudinal BML size to Longitudinal Full Thickness Cartilage Lesion Area

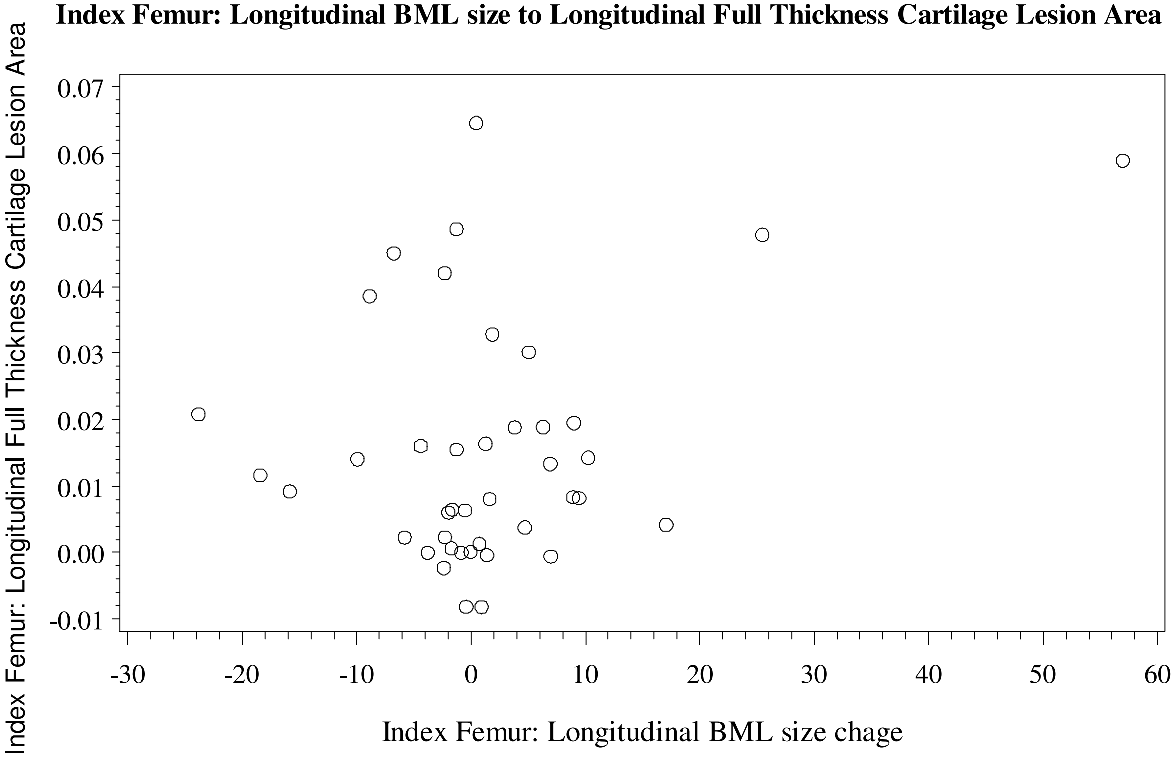

Index Tibia: Longitudinal BML size to Baseline Cartilage Volume

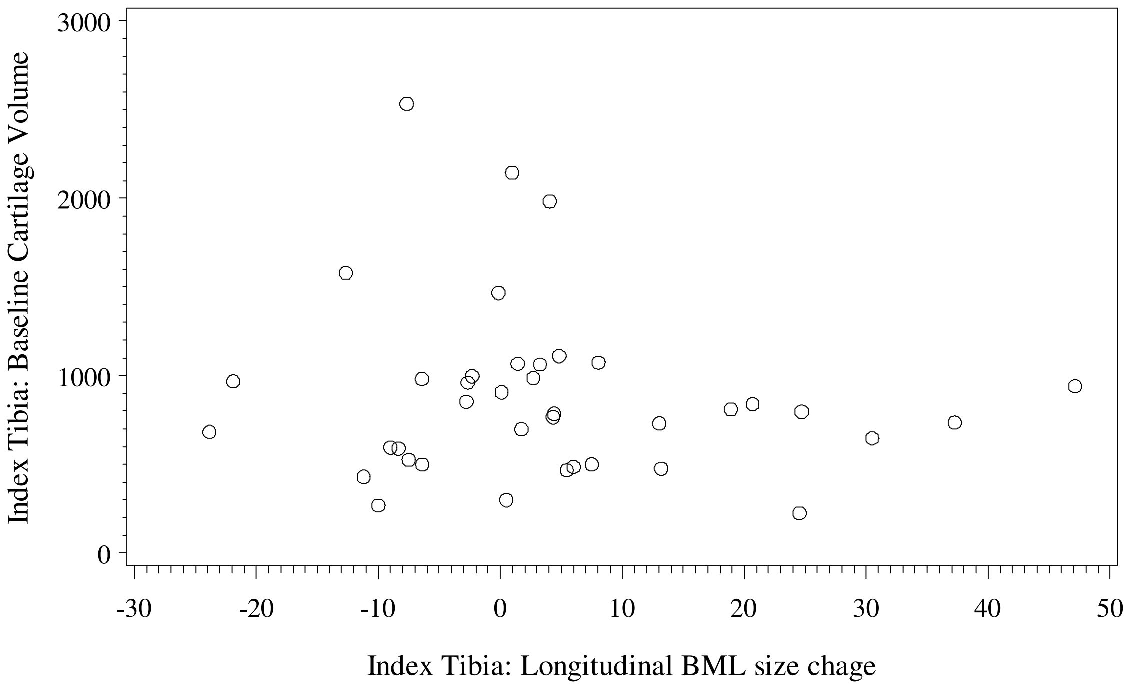

Index Tibia: Longitudinal BML size to Baseline Cartilage Thickness

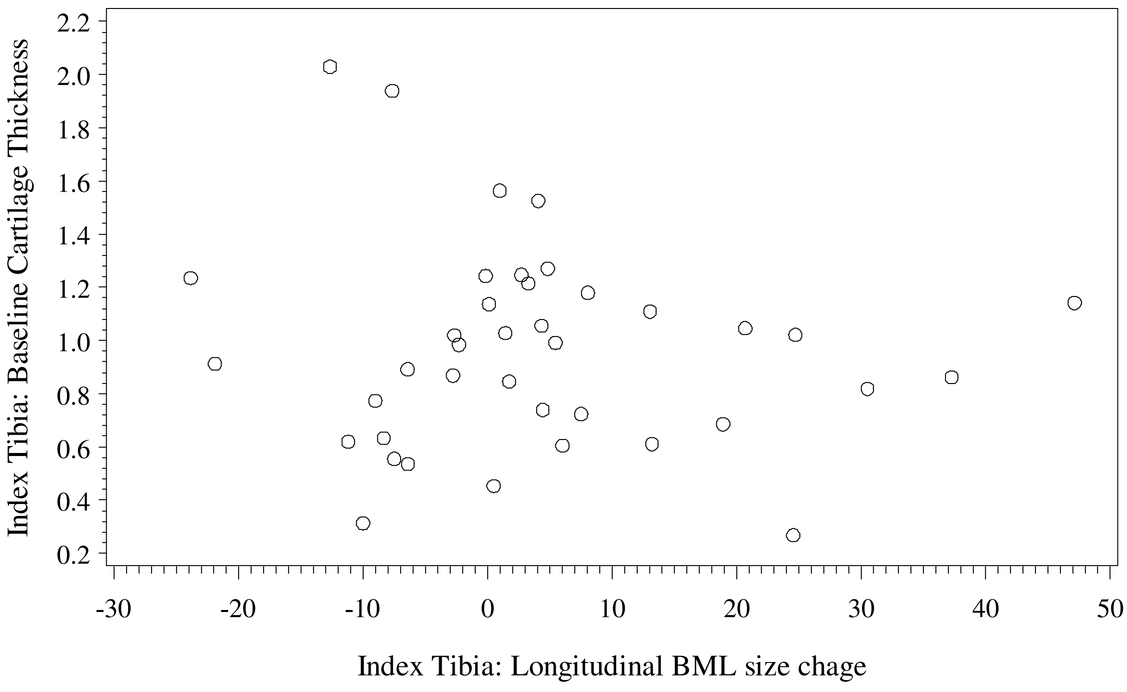

Index Tibia: Longitudinal BML size to Baseline Full Thickness Cartilage Lesion Area

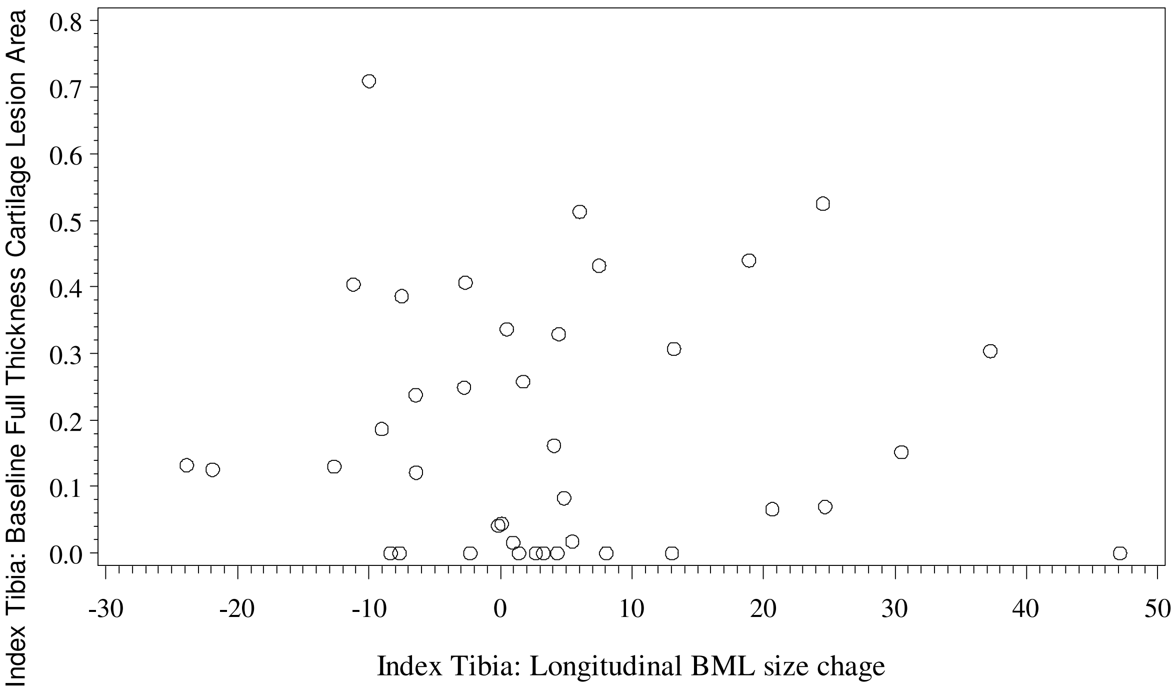

Index Tibia: Longitudinal BML size to Longitudinal Cartilage Volume

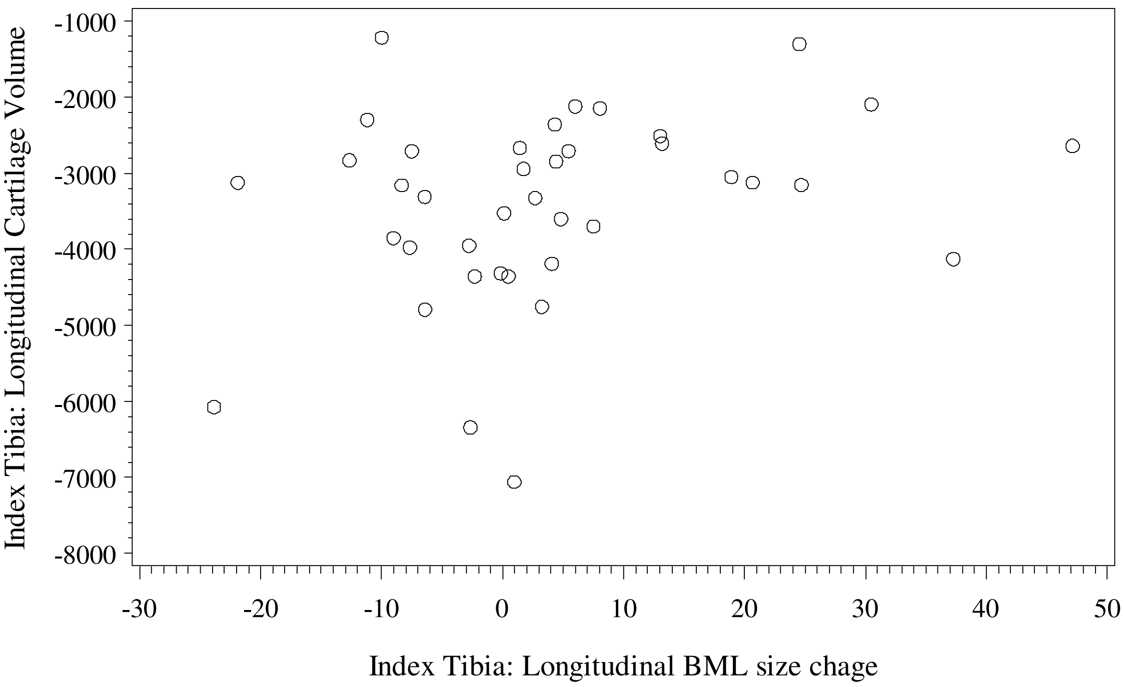

Index Tibia: Longitudinal BML size to Longitudinal Cartilage Thickness

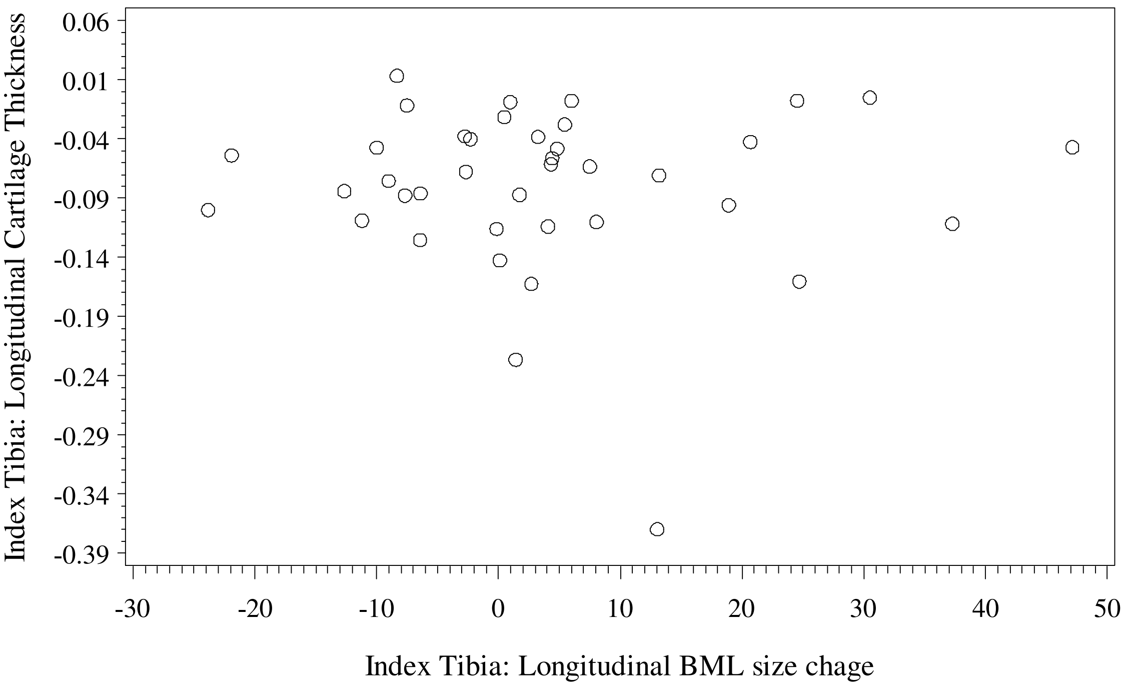

Index Tibia: Longitudinal BML size to Longitudinal Full Thickness Cartilage Lesion Area

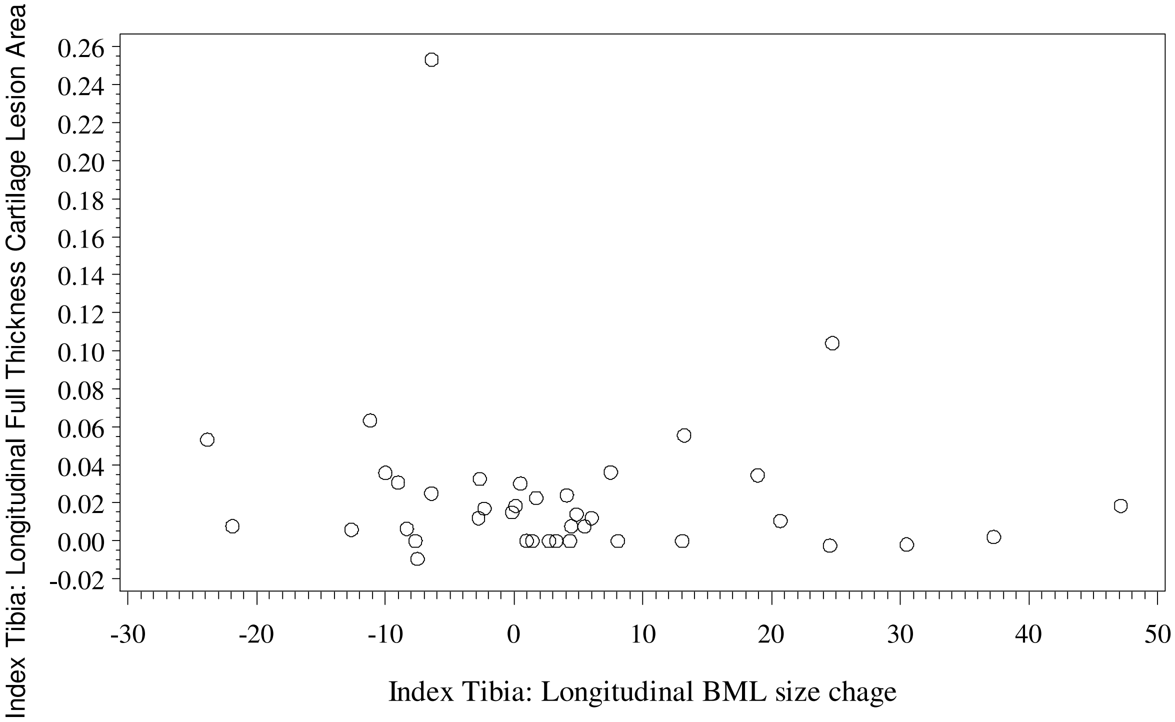

Supplement: Additional File 1 — Intra-regional Scatter Plots. Scatter plots for correlations presented in Table 1 and Table 2 are provided. [file 1471-2474-12-217-S1.PDF]
