# Supplementary material for: Quantitative bone marrow lesion size in osteoarthritic knees correlates with cartilage damage and predicts longitudinal cartilage loss
Source: BMC Musculoskelet Disord. 2011 Sep 30;12:217. doi: 10.1186/1471-2474-12-217 (PMC3190349; doi:10.1186/1471-2474-12-217)
Supplement: Additional File 2 — Additional Table. Within-Region Spearman Coefficients (95% Confidence Intervals) Among Baseline BML and Cartilage Parameters in the Index Compartment. This table demonstrates the correlations between approximate BML volume, cartilage volume, and cartilage thickness among the entire study cohort (n = 107). These correlations were evaluated and determined to be similar between the primary subset (n = 44; Table 2) and entire cohort (n = 107). [file 1471-2474-12-217-S2.PDF]

Additional Table. Within-Region Spearman Coefficients (95% Confidence Intervals) Among Baseline BML and Cartilage Parameters in the Index Compartment

	Femur BML Volume: Baseline (n = 72)	Tibia BML Volume: Baseline (n = 73)	Femur BML Volume: Change (n = 78)	Tibia BML Volume: Change (n = 75)
Baseline				
Cartilage Volume	-0.00 (-0.23, 0.23)	-0.39 (-0.57, -0.17)*	-0.11 (-0.32, 0.12)	-0.14 (-0.35, 0.09)
Cartilage Thickness	-0.06 (-0.29, 0.17)	-0.42 (-0.59, -0.21)*	-0.17 (-0.38, 0.06)	-0.18 (-0.39, 0.05)
2-year Longitudinal Change				
Cartilage Volume	-0.15 (-0.37, 0.09)	0.03 (-0.21, 0.26)	0.08 (-0.15, 0.29)	0.16 (-0.07, 0.38)
Cartilage Thickness	-0.16 (-0.38, 0.07)	-0.14 (-0.36, 0.09)	0.05 (-0.17, 0.27)	-0.02 (-0.25, 0.21)

* statistically significant finding after Bonferroni corrections (16 multiple comparisons; $p < .003$).
